# JAK/STAT signaling is necessary for cell monosis prior to epithelial cell apoptotic extrusion

**DOI:** 10.1038/cddis.2017.166

**Published:** 2017-05-25

**Authors:** Alba Y Torres, Marianne Malartre, Anne-Marie Pret, François Agnès

**Affiliations:** 1 Institute for Integrative Biology of the Cell (I2BC), CEA, CNRS, Université Paris-Sud, Université Paris-Saclay, 91198 Gif-sur-Yvette Cedex, France; 2 Institute for Integrative Biology of the Cell (I2BC), CEA, CNRS, Université Paris Sud, Université, Paris-Saclay, 91198 Gif-sur-Yvette Cedex France; 3 Université de Versailles St Quentin en Yvelines, 78035 Versailles, France

## Abstract

Epithelial cell extrusion is crucial for proper development and tissue homeostasis. High-resolution 3D reconstruction and 4D imaging, combined with genetic analyis, have allowed us to reveal the highly-sterotyped morphogenetic events controlled by JAK/STAT signaling in a developmentally-programmed case of epithelial cell extrusion. Specialized somatic cells, Polar Cells (PCs), are produced in excess and then undergo apoptotic elimination from the follicular epithelium in the *Drosophila* ovary. We show that supernumerary PCs are first systematically enveloped by PC neighbors on all sides, first laterally, then apically in conjunction with highly-reinforced adherens junctions, and finally basally. The PC to be removed thus loses all contact with follicle cells, germline cells and the basement membrane in a process we have called cell ‘monosis’, for ‘isolation’ in Greek. PC monosis takes several hours, and always precedes, and is independent of, activation of apoptosis. JAK/STAT signaling is necessary within the surrounding follicular epithelium for PC monosis. Minutes after monosis is complete, PC apoptotic corpses are formed and extruded laterally within the epithelium, in contrast to the apical and basal extrusions described to date. These apoptotic corpses are engulfed and eliminated by surrounding follicle cells, which are thus acting as non-professional phagocytes. This study therefore shows the non cell-autonomous impact of an epithelium, via JAK/STAT signaling activation, on cell morphogenesis events leading to apoptotic extrusion. It is likely that the use of high-resolution 3D and 4D imaging, which allows for better spatio-temporal understanding of morphogenetic events, will reveal that cell monosis and lateral extrusion within an epithelium are pertinent for other cases of epithelial cell extrusion as well.

Cell extrusion is a process by which single cells are removed from an epithelium. It is crucial for tissue morphogenesis and homeostasis. Its perturbation leads to loss of epithelial integrity and has severe consequences on development and health,^1^ leading to epithelial pathologies, including hyper-immune response in asthma, coeliac disease and irritable bowel syndrome, pathogenic bacterial infection, tumor cell invasion and metastasis. A few studies conducted in various developmental models and in cell culture have described specific cell remodeling events occuring during epithelial cell extrusion. These studies indicate a diversity of extrusion modalities, including either live or apoptotic cell extrusion,^2, 3, 4^ apical or basal extrusion,^5, 6, 7^ and different types of cell-cell contact exchanges.^3^ A role for acto-myosin contraction, which can take place in the cell to be extruded and/or in the surrounding epithelial cells, has also been reported.^8, 9, 10^ Although real-time imaging has been used in some of these studies, high 3D resolution and additional non-induced cell extrusion models are needed to fully comprehend the cell remodeling events occurring at the cellular and tissue levels. In addition, how signaling between cells allows coordination of these events in an epithelium has been addressed in very few cases of cell extrusion.^11, 12^

JAK/STAT signaling is highly conserved between *Drosophila* and mammals, both structurally and functionally,^13^ and its misregulation has important implications in human health.^14, 15^ In the adult *Drosophila* ovary, JAK/STAT activity within the somatic monolayered follicular epithelium surrounding each germline cyst has been shown to control several epithelial morphogenetic events. Polar cells (PCs) located at the anterior and posterior poles of each follicle secrete the cytokine-like Unpaired (Upd), which activates JAK/STAT signaling in the surrounding follicle cells (FCs) throughout oogenesis.^16, 17^ During early oogenesis (stages 2–4), we have previously shown that the number of PCs per pole, initially between 3–6 cells, is reduced by apoptosis to exactly two cells.^18^ We have additionally shown that JAK/STAT activity is required for this process both in PCs and neighboring FCs to trigger the canonical Hid-Diap1-caspase apoptotic pathway to eliminate supernumerary PCs.^19, 20^ PC survival, on the other hand, has been shown to involve Notch signaling.^21^ Reduction of PC number to two cells per follicle pole is crucial for secretion of correct levels of Upd ligand for JAK/STAT signaling-mediated specification of the correct number of border cells later in oogenesis (stages 7–9).^20^ Border cells are responsible for fomation of the micropyle, the sperm entry point into the oocyte, and specification of the correct number of these cells is essential for female fertility.^22, 23, 24^

Here we reveal the precise cell remodeling events occurring prior to PC extrusion from the follicular epithelium, as well as the extrusion and clearance modalities. For this, we used high-resolution 3D image analyses both of fixed tissues and in real-time. The first remodeling event is cell isolation during which the PC to be eliminated is fully enveloped by its PC neighbors, thus becoming isolated from the surrounding environment. We have termed this process ‘monosis’, for ‘isolation’ in Greek. Cell monosis is followed by quick lateral extrusion of PC apoptotic corpses within the follicular epithelium, leading to engulfment and digestion by neighboring FCs. Our results show that JAK/STAT signaling in the follicular epithelium drives PC monosis non cell-autonomously, which constitutes a prerequisite for apoptotic extrusion.

## Results

### PCs to be eliminated first undergo through cell monosis, a process of full envelopment by their PC neighbors

**Figure 1:**
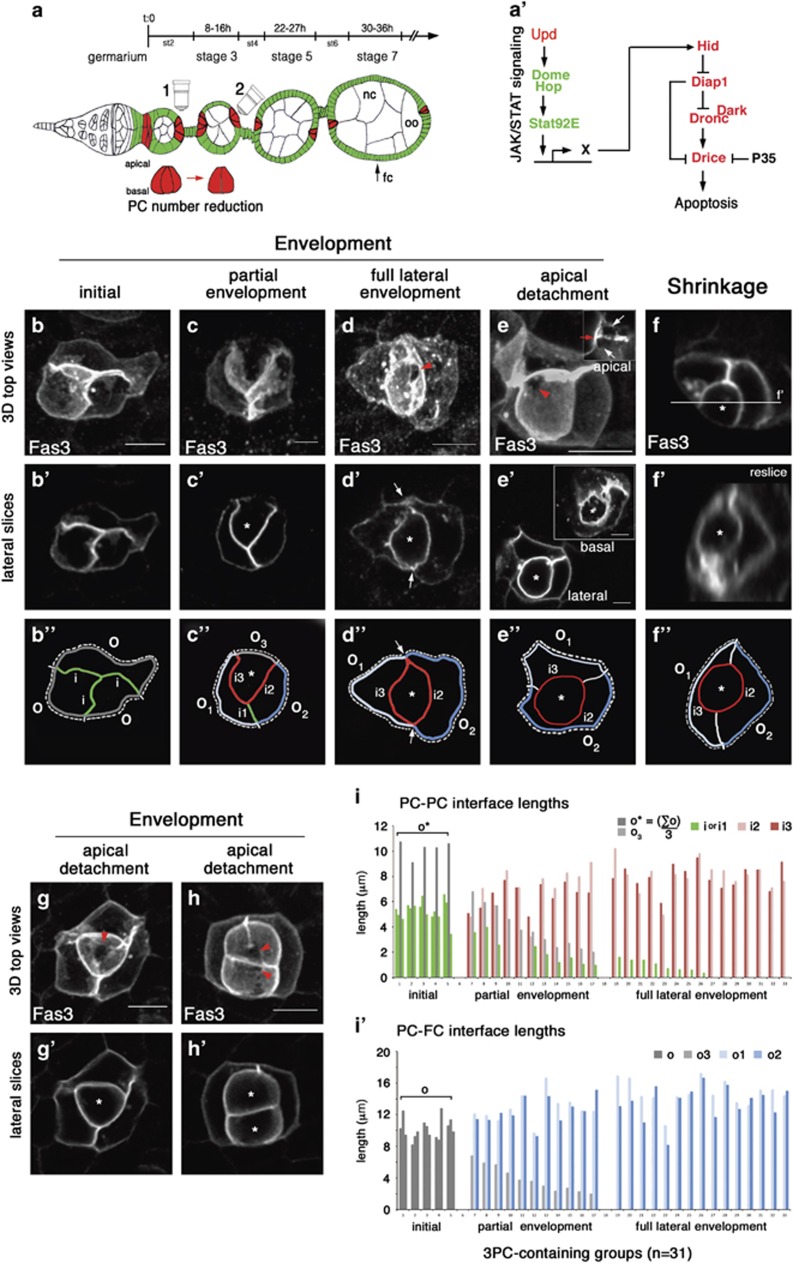
PCs to be eliminated are fully enveloped by adjacent PCs before rounding-up and shrinking. (**a**) Schematic representation of a germarium at the anterior tip of an ovariole and its associated stage 2–7 follicles. In each follicle, a germline cyst composed of nurse cells (nc) and an oocyte (oo) are in white, surrounded by a monolayered epithelium of somatic follicular cells (fc) in green, with apical towards the germline. Follicles are separated from each other by an interfollicular stalk of somatic cells in green. The duration of each follicle stage is indicated above the ovariole.^42^ Polar cells (PCs) within the follicular epithelium at follicle poles are in red. PCs are produced in excess upon follicle formation (up to six cells per follicle pole^18, 19^) and then reduced by apoptosis to two cells at all follicle poles between stage 1 within the germarium (not shown) and stage 5. Microscope objective drawings (1 and 2) indicate the orientation of the views for analysis of PC groups with (1) being called a side view in which apical is oriented to the top for images in all the figures corresponding to this viewpoint and (2) a top view in which the apical-basal axis is parallel to observers viewpoint. (**a')** Schematic representation of the JAK/STAT signaling pathway in *Drosophila* (left) that we have shown to be necessary for activation of the apoptotic cascade within supernumerary PCs.^20^ Unpaired (Upd) is a demonstrated cytokine-like ligand that binds the Domeless receptor (Dome) thereby recruiting the sole *Drosophila* Janus Kinase or JAK, called Hopscotch (Hop), and activating the sole *Drosophila* transcription factor STAT, called Stat92E. Via an as yet uncharacterized mechanism (X), JAK/STAT signaling allows transcriptional activation of the IBM (IAP-binding Motif)-containing pro-apoptotic factor Hid, which leads to downregulation of the anti-apoptotic factor Drosophila inhibitor of apoptosis protein 1 (Diap1) and activation of the initiator caspase Dronc, requiring the activity of its co-factor Dark/Apaf1, and of at least one executing caspase Drice.^20^ Caspase activity can be blocked by the Baculovirus protein p35. (**b–e**) 3D reconstructions of confocal stack top views (see objective 2 in **a**) of groups of 3 PCs immunostained specifically for the membrane protein Fasciclin 3 (Fas3). A putative temporal sequence of events is ordered from (**b–e**) as indicated at the top of the columns from initial, partial envelopment, full lateral envelopment, to apical detachment states (see Supplementary Movie 1). Red arrowheads (**d,e**) point to the limit between the subapical domain of enveloped PCs and the apical domain devoid of Fas3. The inset in (**e**) presents an apical lateral slice of the corresponding 3D reconstruction indicating the contact between the two PCs that have covered the enveloped PC apically as evidence by high Fas3 accumulation (red arrowhead) and the rest of the apical perimeter of these two cells with lower levels of Fas3 (white arrows). (**b'–e'**) A lateral slice of each 3D projection in (**b–e**). Asterisks indicate the PCs being enveloped by the two other PCs (**c'–e'**). The inset in (**e'**) presents a lateral slice of the corresponding 3D reconstruction indicating the basal domain of the enveloped PC (asterisk), which has not detached as yet. (**f**) Confocal side view of a group of 3 PCs (see objective 1 in **a**) showing a round and shrunk PC in the group marked with an asterisk and (**f'**) a 3D reslice of confocal stack corresponding to (**f**), indicating that the round, shrunk PC (asterisk) is enveloped by its two PC neighbors. (**b"–f"**) Drawings of panels (**b'–f'**) indicating PC-PC interfaces (**i** in green, for the initial state and for the other states, i1 in green between the two surviving PCs, i2 and i3 in red between the PC being enveloped and the two enveloping PCs) and PC-FC interfaces (o in grey for the initial state, o1 and o2 in light and dark blue for the two enveloping PCs and o3 in grey for the PC being enveloped). Arrows in (**d"**) point to the positions at which the i1 and o3 interfaces have disappeared as compared to (**c"**). (**g,h**) 3D reconstructions of confocal stacks of 4 PC-containing groups. In (**g**), one supernumerary PC is fully enveloped laterally while the second one is in an initial state indistinguishable from the other two PCs. In (**h)**, both supernumerary PCs are fully enveloped laterally by the other two PCs. Red arrowheads (**g,h**) point to the limit between the subapical domain of enveloped PCs and the apical domain devoid of Fas3. (**g',h'**) Single confocal lateral slices from the 3D reconstructions in (**g,h**). Asterisks indicate the enveloped PCs. Scale bars: 5 *μ*m except for (**e'**) 2.5 *μ*m. (**i,i'**) Quantifications of PC-PC and PC-FC interface lengths using the symbol and color codes presented in (**b"–e"**). The 3PC-containing groups at the three states, initial, partial envelopment, and full lateral envelopment (*n*=31) were ordered according to the decrease in the o3 PC-FC interface of the supernumerary PC being enveloped **(i,i')**. In the initial state, the supernumerary PC to be eliminated cannot be distinguished from the other two PCs so the PC-PC and PC-FC interfaces lengths (green bars) and PC-FC (grey bars) are given the same color code. In the initial state, the PC-PC interface lengths were averaged to simplify the representation in the graph (o* in **i**). Comparison of the average length distributions of the PC-PC interfaces (i *versus* i2, i *versus* i3) and the PC-FC interfaces (o *versus* o1 and o *versus* o2) between the initial and each of the two othere states (partial and full lateral envelopment) show significant statistical differences, *P*=8.57^e-05^ and *P*=1.55^e-04^, respectively

Cell remodeling events occurring during elimination of supernumerary PCs were characterized using high-resolution confocal images acquired from top views (objective 2, Figure 1a) and processed for 3D renditions. Follicles were immunostained for Fascilin 3 (Fas3), a marker of lateral membranes that is upregulated in PCs. The analysis was performed primarily on groups of three PCs since these were the most frequent. Specific configurations were systematically recovered, which allowed us to propose a stereotyped sequence of remodeling steps. In the ‘initial’ state, the three PCs presented radial symmetry (Figures 1b and 3d animation in Supplementary Movie 1). PC-PC and PC-FC interface lengths at the mid-lateral level (Figure 1b') were similar for the three PCs (Figures 1i and i'). No morphological criterion was thus observed in this state to identify the supernumerary PC fated to die. In the ‘partial envelopment’ state, two PCs were partially wrapped around the third PC, which was thus no longer equivalent to the other two (Figures 1c–c" and Supplementary Movie 1). Partial envelopment was illustrated by the fact that the two enveloping PCs had longer PC-FC contacts (o1 and o2) compared to that of the PC being enveloped (o3) (Figures 1c–c" and i"). Also, the contact length between the two enveloping PCs was shorter than initially (i1), whereas that between these cells and the PC being enveloped was the same length or longer (i2 and i3)(Figures 1c–c" and i'). In the ‘full lateral envelopment’ state, the central PC had no lateral contacts with FCs (Figures 1d–d" and Supplementary Movie 1). The two outer PCs completely surrounded it and made a new contact with each other as evidenced by formation of a new vertex (Figures 1d' and d", arrows and i'). In the ‘apical detachment' state, the laterally enveloped PC was fully covered apically by the two enveloping PCs (Figure 1e, inset-white arrows), which thus form a new contact (Figure 1e, inset-red arrow and Supplementary Movie 1) In this state, the enveloped supernumerary PC had no contact with the germline cyst apically. In this configuration, a basal footprint of the PC being enveloped was observed, indicating that basal envelopment had not occurred (Figure 1e' -inset, Supplementary Figures 1b and c). At the ‘shrinkage’ stage, the fully embedded PC presented a reduced size and spherical shape (Figures 1f and f'). This configuration was rarely observed, suggesting that the process between apical detachment and elimination of supernumerary PC occurs quickly. Groups of four or more PCs, although much less represented, were also analyzed in the same way and images of supernumerary PCs enveloped by PC neighbors were also recovered (Figures 1g and Supplementary Figures 1b and b'). Taken together, these data suggest a stereotyped sequence of cell remodeling events whereby supernumerary PCs become progressively fully enveloped by their neighboring PCs. We have named this process cell ‘monosis’ (‘isolation’ in Greek) since supernumerary PCs become fully isolated from other cells within the tissue, namely follicular epithelial cells and germline cysts (Model in Figure 8a.

### Adherens junctions are reinforced during supernumerary PCs monosis

**Figure 2:**
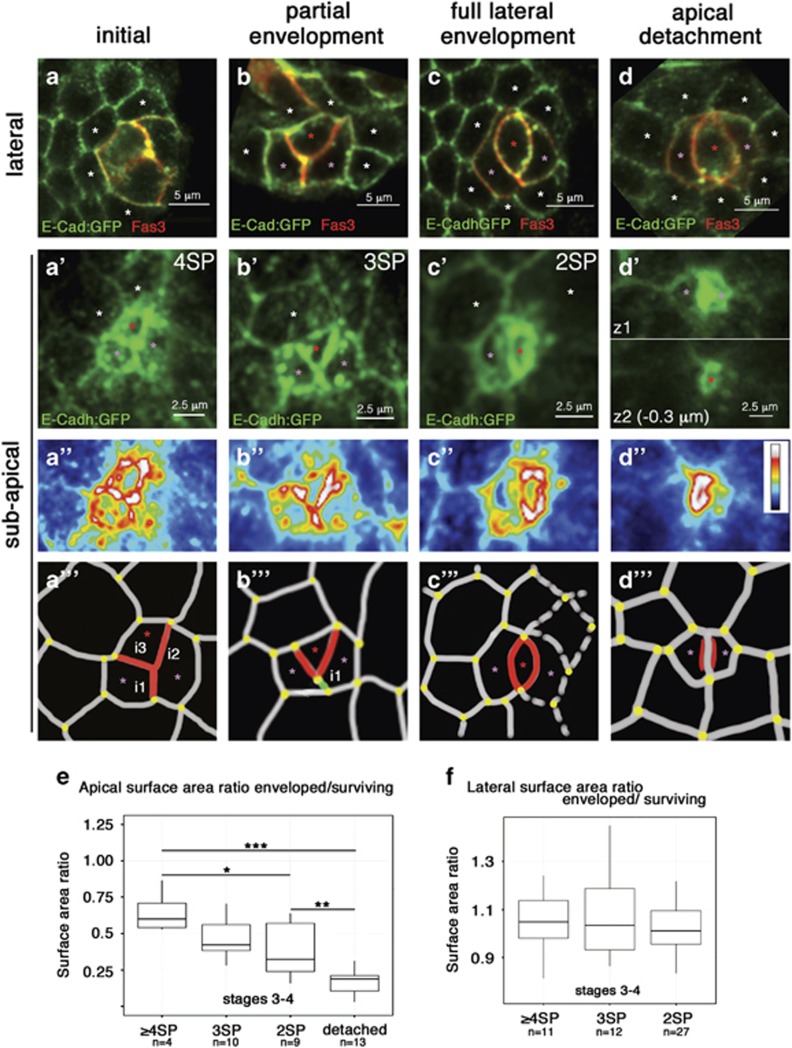
Envelopment of supernumerary PCs involves progressive apical constriction and loss of FC contacts associated with reinforced AJs. (**a–d**) Stage 3–4 follicles carrying the ECad:GFP knockin transgene were immunostained for Fas3 and GFP. Projections are presented of confocal slices through the lateral domain of three PC-containing groups from top views (see objective 2 in Figure 1a), which indicate the different states of envelopment. White asterisks indicate the FCs in contact with PCs. Supernumerary PCs which are enveloped by other PCs are indicated by red asterisks and the enveloping PCs by pink asterisks. (**a'–d'**) Projections of confocal slices of the same PC groups as in (**a–d**), which presented the highest levels of ECad:GFP in the PC being enveloped (corresponding to the subapical domain of this cell). ECad:GFP in the subapical domain of the PC being enveloped delimited 4-sided, 3-sided, and 2-sided (or lens-shaped) polygons (4SP,3SP,2SP- red asterisks). Thus, at the different states, a different number of cell contacts with FCs (white asterisks) is observed. In d', z1 presents a subapical slice of the two enveloping PC forming a new contact, while z2 is a slice that is 0.3 *μ*m lower than z1 in which the presence of the lens-shaped ECad:GFP domain of the supernumerary PC indicates that it has detached apically. (**a"–d"**) Images in (**a'–d'**) panels treated with a royal color code. The calibration bar is shown in (**d"**); blue indicates the lowest and white the highest intensities. (**a"'–d"'**) Schematics of images in (**a'–d'**). The different PC/PC contacts (i1–i3) are drawn in red except for i1 in (**b"**), which is in green and displays a shorter length than in (**a"**). PC-FC and FC-FC outlines are in grey. Yellow dots materialize all vertices. During apical detachment (**d"**), the most apical portion of the cell to be eliminated (red lines) is beneath the surface of the two enveloping PCs. (**e,f**) Boxplots of ratios of the apical and lateral surface areas, respectively, of the supernumerary PC being enveloped over those of its adjacent PCs as a function of the state of envelopment. *n*=the total number of groups of 3 PCs analyzed per point. *P=0.01206, **P=0.00595, ***P=0.001

We used an E-Cadherin:GFP (ECad:GFP) knockin line to visualize subapical adherens junctions (AJs) and characterize cell remodeling events occurring in the apical domain during PC envelopment. At different envelopment states, ECad:GFP accumulation was higher at subapical PC-PC interfaces than at equivalent PC-FC and FC-FC interfaces (Figures 2a'–d"'), with the highest levels found between the PC being enveloped and its two PC neighbors (Figures 2a"–d" and 3d animation in Supplementary Movie 2). This is thus the first molecular marker for the PC apoptotic fate before significant cell remodeling has occurred (Figure 2a'–a"', red asterisk). The same result was obtained using immunostaining with antibodies directed against ECad (Supplementary Figures 1a and a'). Armadillo and Bazooka, respectively the *β*-catenin and Par3 *Drosophila* homologs, were also enriched specifically in the subapical domain of the PC being enveloped, precisely at the two PC interfaces (Supplementary Figures 1c–g). AJs in the subapical domain are therefore reinforced between PCs during the envelopment process. In contrast, when the fully embedded supernumerary PC was at the shrinkage stage, ECad immunostaining was lower and the protein was homogeneously distributed throughout the cortex of the dying cell (Supplementary Figures 1b and b'). Our data thus indicate that remodeling of the apical domain during supernumerary PC cell remodeling involves the formation of reinforced AJs with neighboring PCs prior to apical detachment and shrinkage (see model, Figures 8a and b).

### Both apical and basal constrictions occur during supernumerary PCs monosis

PC subapical, lateral and basal surface areas were analyzed during PC envelopment, using ECad:GFP and Fas3 markers. The subapical domain area, but not that of the lateral domain, showed significantly lower measures for the PC to be eliminated compared to its surviving PC neighbors, correlating directly with a lower number of FC neighbor contacts (Figures 2e and f, respectively). The basal cell area was also smaller both at the full lateral envelopment and apical detachment stages than at the initial and partial envelopment states (Figure 1d', inset and data not shown). Our results hence suggest that supernumerary PC monosis involves specific apical and basal constrictions prior to detachment and shrinkage (Figures 8a and b).

### The number of follicle cell contacts is reduced during supernumerary PC monosis until no contacts with FCs remain

We next determined the number of PC-FC contacts during PC monosis using ECad:GFP. At the initial state, each PC formed subapical ECad:GFP^+^ contacts with two or more FCs, defining four-, five- or six-sided polygons (4SP, 5SP, 6SP, Figures 2a'–a"' and data not shown). The PC at the partially enveloped state formed subapical contacts with one or two FCs defining domains with 3SP or 4SP shapes, respectively (Figures 2b–b"'). At the fully laterally enveloped state, the enveloped PC, no longer in contact with FCs, formed a 2SP (lens-shaped) subapical domain at the contact with the enveloping PCs (Figure 2c–c"'). Upon apical detachment, as evidenced by the new subapical contact formed between the enveloping PCs (Figure 2d'), the detaching PC maintained a 2SP subapical domain, which had sunken to a lower level (Figure 2d'). At the lateral domain of the supernumerary PC at different states of envelopment, similar reudction in the number of FC contacts was observed (Figures 2a–d).

### Real-time imaging reveals that PC monosis requires several hours, whereas apoptotic extrusion occurs quickly

**Figure 3:**
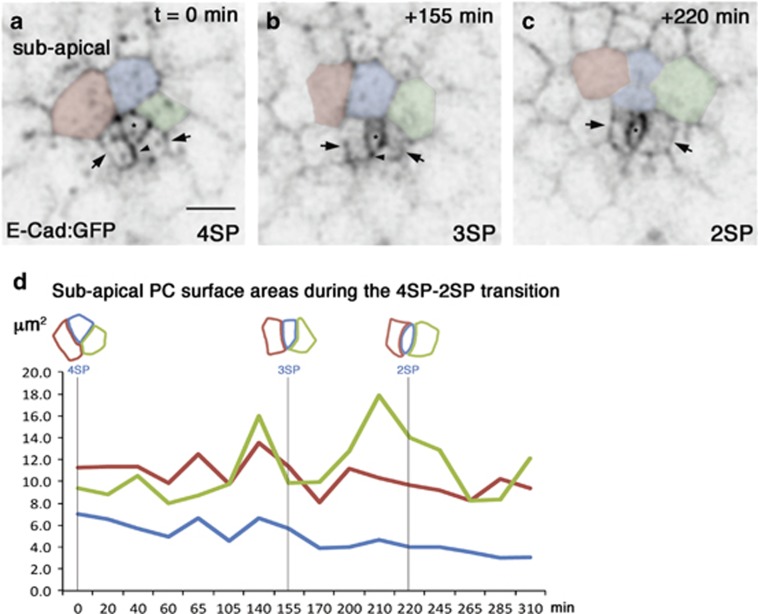
Real-time imaging of the early steps in supernumerary PC envelopment. (**a**–**c**) Three different frames of a 5 h live time-lapse confocal image acquisition of a group of 3 PCs grey scale). *t*=0 corresponds to >2 h from the beginning of image acquisition of the cultured follicle. PCs are identified by higher ECad:GFP accumulation at a subapical level compared to their FC neighbors (as observed from an objective 2 position, see Figure 1a). The PC being enveloped is indicated with an asterisk and the two enveloping PCs with arrows. Three FCs have been false colored in pink, blue and green. The PC being enveloped first has two FC and two PC interfaces (**a**, 4SP), then one FC and two PC interfaces (**b**, 3SP) and finally only two PC interfaces (**c**, 2SP or lens shape). The arrowheads point to the i1 PC-PC interface (see Figure 1c"), which progressively reduces until disappearing. Scale bar: 5 *μ*m. (**d**) Graph plotting subapical cell surface areas as delimited by ECad:GFP in the supernumerary PC being enveloped (blue) and surrounding PCs (red and green) at different times of the Supplementary Movie 3 corresponding to (**a–c**)

Real-time imaging was conducted on stage 3–4 follicles from ECad:GFP knockin flies, also carrying a Pleckstrin Homology Domain:CherryFP (PH:ChFP) transgene. This allowed us to characterize PC remodeling events at the AJs level and to determine overall cell shape changes of both PCs and surrounding FCs. Imaging of the entire volume of PC groups over several hours enabled us to confirm the sequential order of remodeling events we had deduced from fixed-tissue analysis. In addition, they also provided the timing for the different phases. The initial to full lateral envelopment stages revealed transitions from the 4SP to the 2SP shapes made by subapical Ecad:GFP encompassing a period of about four hours (Figures 3a–c and Supplementary Movie 3; *n*=2 films). During this period, the supernumerary PC subapical surface areas progressively decreased, while that of the two enveloping PCs exhibited oscillating size changes, but no significant overal reduction in size (Figure 3d and Supplementary Movie 3).

**Figure 4:**
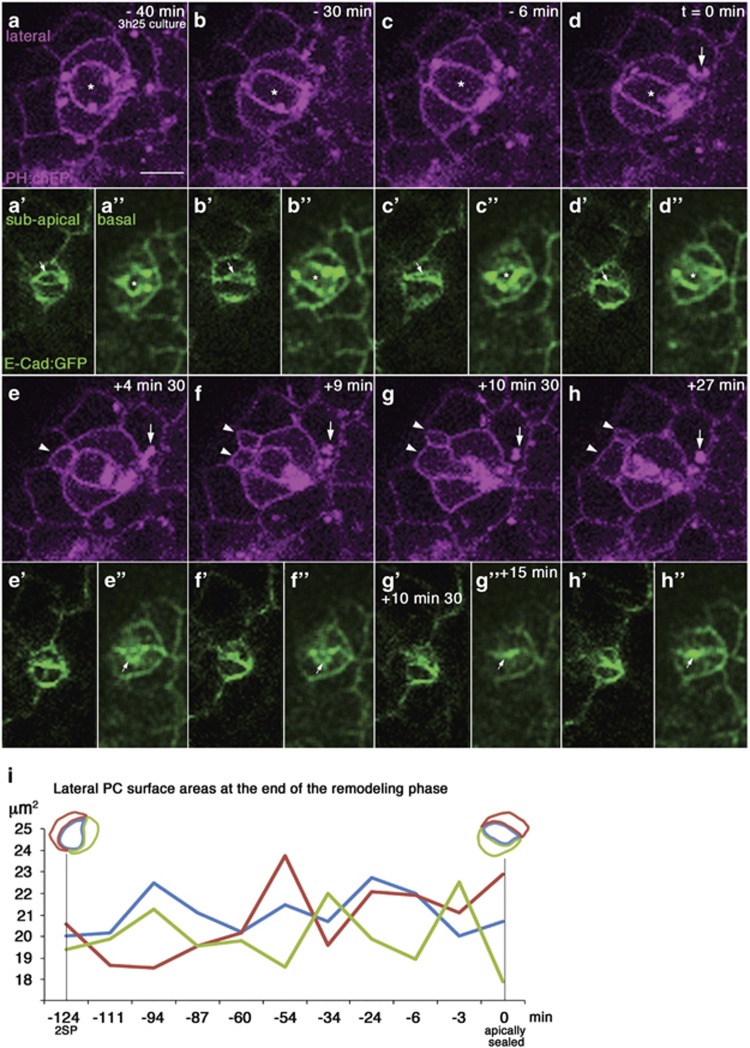
Real-time imaging of lateral expulsion of PC apoptotic corpses. (**a–h**) Frames of covering 67 min of a >10 h time-lapse confocal image acquisition of a cultured stage 3 follicle expressing ECad:GFP and PH:chFP. Lateral, subapical and basal slices (**a–h, a'–h'** and **a"–h"**, respectively) are presented through a group of 3 PCs within the follicular epithelium using the objective 2 viewpoint (see Figure 1a). (**a**) The supernumerary PC that is fully laterally enveloped by two adjacent PCs (asterisk) and apically detached (data not shown) presents a 2SP or lens-shaped subapical domain as delimited by ECad:GFP (**a'**), as in Figure 2d'z2 and Supplementary Figure 1c"z2. The frame corresponds was taken 3 h 25 min after the beginning of image acquisition of the cultured follicle. The scale bar represents 5 *μ*m and is the same for all the images. (**a'**–**d'**) Apical surface reduction of the supernumerary PC (arrows) is observed until sealing of this domain into a line (**d'**), which was considered as time *t*=0 min. (**e**–**h**) At *t*=+4 min30 s, lateral expulsion of a first apoptotic corpse within the follicular epithelium is observed (**e-**arrowhead). At few minutes later, at *t*=+9 min, a second apoptotic corpse is expulsed (**f**-bottom arrowhead) and by *t*=+27 min the enveloped supernumerary PC has collapsed and its contents is all within the expulsed apoptotic corpses (**h**). (**a"–h"**) Full basal cell surface area sealing was also observed (compare **a"–f"** to **g",h"**, arrows) and it occurred after complete apical cell surface reduction and the beginning of extrusion of apoptotic corpses (**g"**, *t*=+15 min). (**i**) Graph plotting lateral cell surface areas of the supernumerary PC (blue) and surrounding two PCs (red and green) from 127 min before apical detachment (2SP) to apical detachment (or sealing)

Real-time imaging also allowed visualizing the extrusion events occurring upon completion of PC monosis, which had not been possible using fixed-tissue analysis. Groups of three PCs at the apically-detached stage but still possessing a 2SP-shaped ECad:GFP-enriched subapical structure were filmed, revealing that the this structure did not change during a period of more than four hours (Figures 4a–a' and Supplementary Movie 4; *n*=2 films). The three PCs exhibited similar lateral dynamic cell shape changes and area size oscillations during this time period, without an overall decrease in size (Figures 4a–d and i). Finally, the enveloped PC completed subapical surface area reduction or sealing (Figure 4d'), Strikingly, within only a few minutes (3–6 min) after sealing, protuberances from the enveloped PC were extruded from between the two enveloping PCs, laterally within the follicular epithelium (Figures 4e–h, arrowheads). They grew in size while remaining in continuity with the enveloped PC, which concomitantly reduced and eventually collapsed (Figures 4e–h and Supplementary Movie 4). During this process, the basal surface area reduced in size and was finally sealed a few minutes after extrusion of apoptotic corpses had already begun (Figures 4a"–h", asterisks and arrows). These results indicate that, while cell monosis was long (>4 h), the extrusion phase began just minutes after full reduction and collapse of the subapical domain, suggesting that completion of envelopment may trigger the apoptotic phase.

### Real-time imaging shows that PC apoptotic corpses are extruded laterally within the epithelium and engulfed by surrounding follicle cells

**Figure 5:**
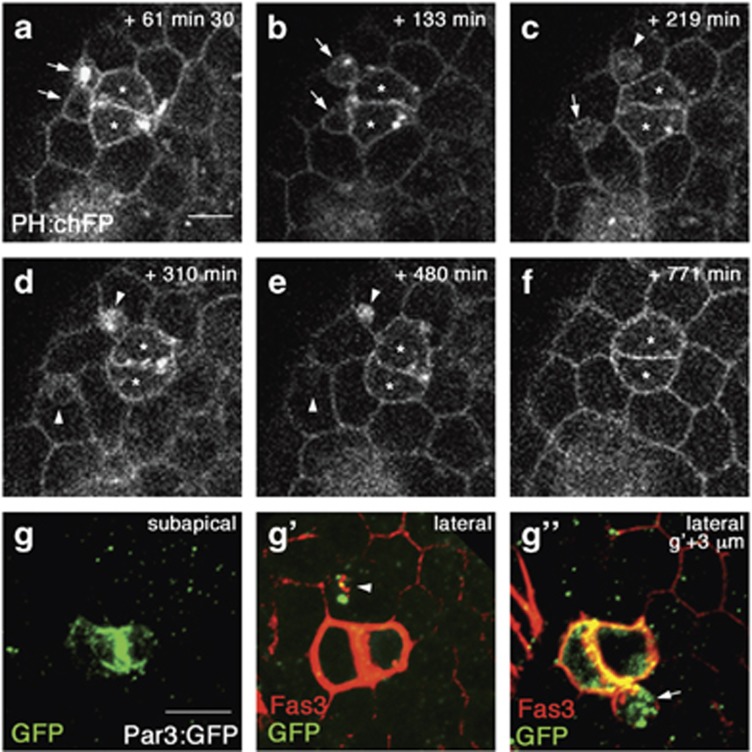
Apoptotic corpses are engulfed and digested by neighboring FCs. (**a**-**f**) Frames of time-lapse confocal image acquisition of a cultured stage 3 follicle expressing PH:chFP beginning 61 min30 after apical detachment of the supernumerary PC and covering the following 710 min. Lateral slices are presented through a group of 3 PCs within the follicular epithelium using the objective 2 viewpoint (see Figure 1a). Asterisks indicate the two surviving PCs, arrows mark the two expulsed apoptotic corpses between FCs and arrowheads the apopotic corpses once engulfed by a neighboring FC. Scale bar: 5 μm. **(d**-**f)** The two engulfed apoptotic corpses decrease in size within the FCs and finally disappear (between **e** and **f**). **(g–g")** Subapical, lateral and lateral +3μm towards basal confocal image projections taken from the viewpoint of objective 2 (see Figure 1a) of a follicle pole from fixed tissue (*upd-Gal4>UAS-par3:GFP*) immunostained for Fas3 and GFP to visualize Par3. Scale bar: 5 μm. (**g**') Debris from a PC apoptotic corpse, marked by Fas3 and Par3:GFP is detected inside a FC (**g'**, arrowhead) adjacent to a PC pair marked by strong Fas3 membrane accumulation. (**g’’**) An extruded apoptotic corpse is found at a more basal level marked with both Fas3 and Par3:GFP (arrow)

Real-time imaging also revealed for the first time the fate of the PC apoptotic corpses. Once laterally expulsed within the follicular epithelium, these are engulfed by neighboring FCs (Figures 5a–f, Supplementary Movie 5; *n*=4 films). Some apoptotic corpses were fully internalized by immediately adjacent FCs within about 1 h of extrusion (Figures 5a–f, top arrows and arrowheads), while others migrated past the adjacent FCs to be engulfed, within about 2 h, by a FC not in contact with the PC group (Figures 5a–e, bottom arrows and arrowheads). In both cases, the complete elimination of engulfed apoptotic material took several hours (Figure 5f). PC apoptotic material engulfed by FCs was also observed in fixed tissues using a Par3:GFP fusion protein specifically expressed in PCs. Par3:GFP accumulated in not as yet engulfed Fas3^+^ apoptotic corpses (Figure 5g"-arrow) and in globular Fas3^+^ apoptotic remnants present in the cytoplasm of adjacent FCs (Figure 5g'- arrowhead). Therefore, FCs appear to be able to function as phagocytes to eliminate PC apoptotic corpses.

### PC monosis does not require the activation of the apoptotic cascade

**Figure 6:**
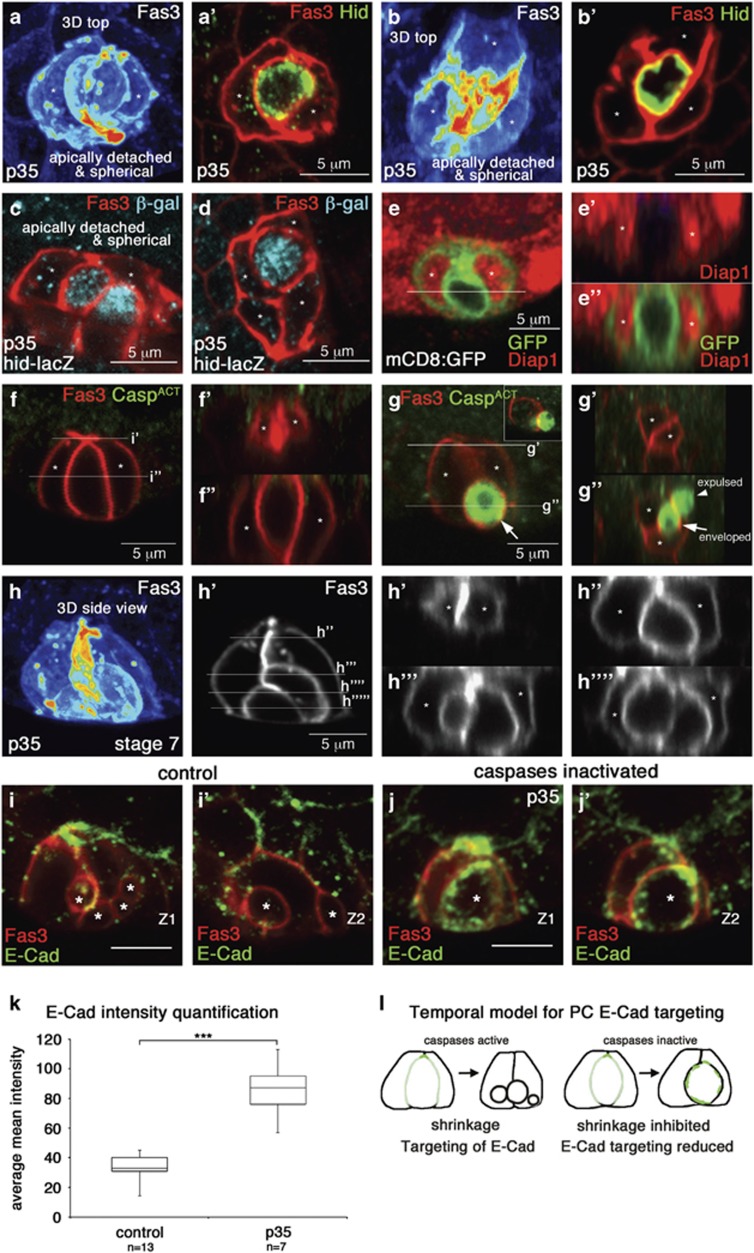
Molecular and functional evidence for a PC apoptotic phase occurring after PC envelopment and apical detachment phases. (**a**–**h**) 3–5 PC-containing groups immunostained for the PC-specific membrane protein Fas3. 3D reconstruction top views of stacks of confocal images (**a,b**-objective 2 Figure 1a) and corresponding lateral slice projections (**a',b'**), confocal projection from top view (**d**, see objective 2 in Figure 1a) and confocal projections (**c,e–g,h'**) and 3D reconstructions (**h**) from side views (see objective 1 Figure 1a) and corresponding XZ reslices (**e',e",f',f",g',g",h'-h""**) at the levels indicated by the horizontal bars in (**e–g,h'**). **(a–h)** Asterisks indicate PCs that are participating to envelopment of a supernumerary PC. (**a**–**d,h**) All PCs express the casapse inhibitor p35 using the *upd-Gal4* and *UAS-p35* transgenes. (**a',b'**) show Hid immunostaining, (**c,d**) *hid-lacZ* transcriptional reporter expression, (**e–e"**) specific absence of Diap1 immunostaining and (**f–f",g–g"**) caspase activation, only in enveloped supernumerary PCs. (**h–h""**) A stage 7 follicle expressing the caspase inhibitor p35 in PCs (presence of the *upd-Gal4* and *UAS-p35* transgenes) exhibits prolonged survival of supernumerary PCs that have already been enveloped and are round and shrinking. **(i,j)** Early stage control (*upd-Gal4/UAS-mcD8:GFP*) and *upd-Gal4/UAS-p35/UAS-mcD8:GFP* follicles with>2PCs immunostained for Fas3 and endogenous ECad. Two different lateral projections (Z1 and Z2, objective 1 side view in Figure 1a) for each PC group is shown (**i,i',j,j'**). The supernumerary PCs at different states of rounding-up and shrinking are indicated with asterisks and these show low levels of ECad in the control compared to the high levels in the caspase-inactivated case. (**k**) Box-plots of ECad mean intensity in control apoptotic (average diameter: 2.9 *μ*m) and p35-expressing blocked (average diameter: 4.7 *μ*m) supernumerary PCs. Mean intensity was calculated for each cell or corpse as the average of ECad signal intensity within the area delimited by Fas3 staining in all the slices of the confocal image stacks. Genotypes are the same as in (**i,j**). ***P=5.05e-06. (**l**) Schematic model showing temporal caspase-dependent targeting of ECad during the apoptotic phase. In the control situation to the left, the supernumerary PC being enveloped is in the middle with its interfaces with the two other PCs drawn in dark green at the level of the subapical AJ and in light green at the lateral-basal interfaces. ECad is much lower once the supernumerary PC has been remodeled, and is rounding up and shrinking. Depletion of caspase activity with p35 blocks at the rounded-up and shrinking state and ECad levels remain high. Therefore, ECad may be a target of caspase-dependent degradation only at the late apoptotic phase

We previously reported that supernumerary PCs die through apoptosis via the transcriptional upregulation of *hid* and consequent posttranslational downregulation of Diap1, leading to caspase activation.^19^ In the present study, we wished to determine when the apoptosis cascade was activated with respect to the remodeling steps of the PC extrusion process. We found that both Hid protein and a *hid-lacZ* transcriptional reporter transgene were detected only when supernumerary PC monosis was already complete (Figures 6a–d,d' and animation in Supplementary Movie 6). This was also the case for Diap1 protein downregulation (Figure 6e–e") and caspase activation (Figures 6f–f" and g–g"). The use of four different markers therefore indicated that the apoptosis cascade was active only after the supernumerary PC was fully enveloped.

Next, we inhibited caspase activity by expressing p35 specifically in PCs. As previously reported, under these conditions many follicle poles exhibited more than 2PCs after stage 5 (63%, *n*=611 compared to 1% in controls, *n*=658). We found that many of these 3PC and 4 PC groups (52%, *n*=458 and 59%, *n*=88, respectively) contained supernumerary PCs that had already rounded-up and shrunk (Figure 6h–h"", Supplementary Figure 3a–c). Therefore, inhibiting caspase activity with p35 expression significantly blocked the late apoptotic elimination stages.

**Figure 7:**
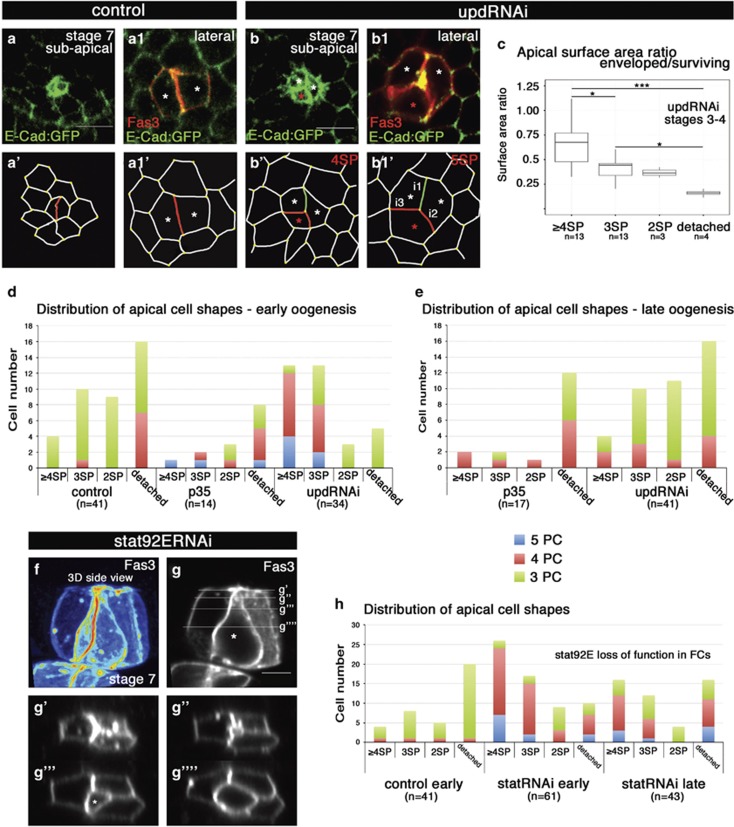
JAK/STAT signaling is necessary for the supernumerary PC remodeling phase leading to apoptosis. Subapical (**a**,**b**) and lateral (**a1,b1**) slices from confocal image stacks taken from the top view (see objective 2 in Figure 1a) for control and *upd*-RNAi expressing stage 7 follicles immunostained for the PC-specific membrane protein Fas3 and an ECad:GFP fusion protein from a knockin transgene. These images are schematized in (**a',a1',b',b1'**) with the PC-PC contacts indicated in red and green and denoted i1–i3 as in Figure 1, while PC-FC and FC-FC contacts are in grey. At stage 7, the control follicle presents a pair of PCs (asterisks **a1,a1'**), while at this same stage, the *upd*-RNAi follicle presents a group of 3 PCs with the supernumerary PC (red asterisk) just beginning to be enveloped at the 4SP state subapically (**b,b'**) and 5SP state laterally (**b1,b1',** enveloping PCs are indicated by white asterisks). Scale bars: 5 *μ*m. (**c**) Box plots of ratio of the subapical surface area as determined by ECad:GFP membrane accumulation between enveloped PCs and enveloping PCs in *upd*-RNAi follicles. (**d,e,h**) Bar graphs presenting the number of supernumerary PCs exhibiting different subapical domain shapes (>=4SP, 3SP, 2SP/lens) as determined by ECad:GFP membrane accumulation from groups of 3, 4 and 5 PCs (green, red and blue bars, respectively). (**d,e**) Control: *upd-gal4/+ECad:GFP/+*. p35: *upd-gal4/+ECad:GFP/UAS-p35*. upd-RNAi: *upd-gal4/+ECad:GFP/UAS-upd-RNAi*. (**h**) Control: *ECad:GFP/+fruitless-gal4/+*. statRNAi: *tub-gal80*^*ts*^*/+ECad:GFP/UAS-Stat92E-RNAi;fruitless-gal4/+.* "early" corresponds to oogenesis stages 2–5 and "late" to stages 6–10. (**f**) 3D reconstruction of a confocal stack of images from a side view (objective 1, Figure 1a) of a group of 4PCs from a stage 7 *stat92E*-RNAi (*fruitless-Gal4>UAS-Stat92E-RNAi)* follicle immunostained for the PC-specific membrane Fas3. (**g**) Single confocal slice from (**f**) with horizontal lines indicating the level of the XZ reslices presented in (**g'–g""**)

Using 3D reconstructions of follicles immunostained for Ecad:GFP, we next quantified the proportion of apical PC shapes (5SP-2SP and detached) observed upon p35-mediated caspase inhibition. At early stages (3–5), there was no obvious difference between p35-expressing and control PC groups (Figure 7d). However, during later stages (6–9), the vast majority of p35-expressing PC groups with supernumerary cells had undergone cell monosis and were detached apically (Figure 7e). Therefore, inhibiting caspase activity did not impede the early monosis and apical detachment processes but blocked the later shrinking process and final elimination of supernumerary PCs. Altogether, these results strongly suggest that the apoptosis cascade, involving Hid, Diap1 and Caspases, is dispensable for the early remodeling steps of the PC group, while being responsible for the later shrinkage and elimiantion steps.

Since AJ components have been shown to be targets of caspase activity,^25^ we compared ECad accumulation in PCs in which caspase activity was inhibited by p35 to that of controls. Whereas p35 expression in PCs did not seem to affect apical ECad accumulation during the early PC remodeling steps (data not shown), it led to abnormally high accumulation of ECad in shrinking supernumerary PCs (Figures 6i–k). This result suggests that AJs are lost in supernumerary PCs during the apoptotic phase in a caspase-dependent manner (Figure 6l).

### JAK/STAT signaling is necessary for PC monosis prior to apoptotic extrusion

As previously reported,^20^ inhibition of JAK/STAT signaling blocks PC apoptosis, resulting in prolonged survival of supernumerary PCs late into oogenesis. We hence wished to determine which step of PC elimination was affected by inhibition of JAK/STAT signaling. Upon RNAi-mediated reduction of the Upd ligand 58% (*n*=397) of PC groups presented more than two PCs after stage 5 compared to 0.5% (*n*=508) in the control. The remodeling stage of the supernumerary cell in groups of three PCs, as evidenced by Fas3 immunostaining, was classified as attached, detached or round using 2D confocal images (Supplementary Figure 3a). From stage 6 onwards, supernumerary PCs, in Upd-deficient follicles, remained attached (59%, *n*=257), while very few proceeded to the shrinking and spherical stage (7%, *n*=257) (Supplementary Figure 3b -right graph). Similar results were obtained in four PC groups (Supplementary Figure 3c) and in follicles depleted for Stat92E specifically in PCs or in FCs (Figures 7f–g"" and data not shown). Together, these results indicate that the activity of the JAK/STAT pathway is necessary for the primary PC remodeling events to occur efficiently.

We next analyzed PC envelopment stages specifically in different contexts of JAK/STAT signaling reduction (*upd-Gal4>upd-RNAi* and *fru-Gal4>Stat92E-RNAi*). Up to stage 5, supernumerary PCs were still in contact with FCs (≥4SP and 3SP states) in a majority of cases (76%, *n*=34), as opposed to controls (37%, *n*=41) (Figures 7d and h). However, progressive reduction in the subapical surface area was still observed upon Upd inhibition (Figure 7c). At later stages, 34% of groups (*n*=41) were found blocked in the remodeling phase in Upd*-*deficient follicles (Figures 7b and 3d animation in Supplementary Movie 7). The distribution was shifted towards the more advanced remodeling states (2SP and apically-detached), resembling the distribution observed in early control oogenesis controls (Figures 7d and e). We observed an even stronger block of primary PC remodeling states (≥4SP and 3SP) upon RNAi-mediated inhibition of *Stat92E* in FCs, both at early and late stages of oogenesis (Figures 7f–h and 3d animation in Supplementary Movie 8). Finally, clonal analysis using two different *stat92E* null alleles also showed that loss of *stat92E* in either PCs or FCs led to prolonged survival of supernumerary PCs blocked during early monosis (Supplementary Figure 2 and data not shown). Altogether these results demonstrate that JAK/STAT signaling is required for the early PC remodeling events occurring during cell monosis.

## Discussion

**Figure 8:**
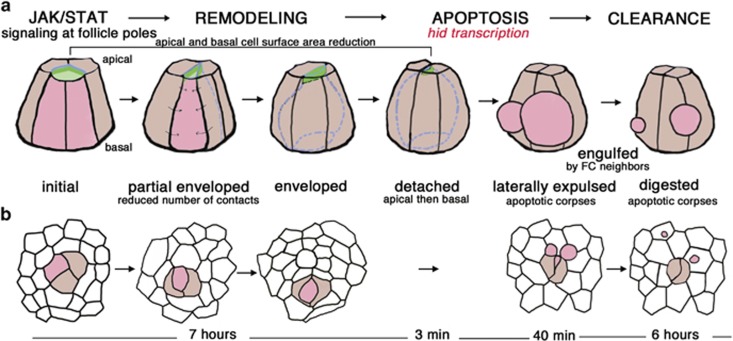
Model for JAK/STAT-mediated remodeling of supernumerary PCs triggering apoptotic elimination of these cells. (**a,b**) Schematic drawings based on results from analysis of fixed and living ovarian follicles. Groups of PCs are shown in which the supernumerary PC to be eliminated is represented in pink or by blue interrupted lines when fully surrounded by its neighboring PCs, the pairs of surviving PCs are in brown and FCs in white. The subapical reinforced AJs composed of ECad, Arm/*β*-Catenin and Par3 are indicated in green. (**a**) 3D side views (see objective 1 in Figure 1a) and (**b**) 2D top views (see objective 2 in Figure 1a) of a group of 3 PCs undergoing the sequential sequence of events leading to elimination of the supernumerary PC with the timing of the steps indicated below. Results indicate that JAK/STAT signaling is necessary for a lengthy remodeling phase (several hours), which leads to full envelopment of the supernumerary PC by its PC neighbors, including apically, such that apical detachment of this cell occurs. Minutes after the subapical AJ domain of the supernumerary PC seals, closely followed by the sealing of the basal domain, the apoptotic phase is activated such that the supernumerary PC rounds-up, shrinks and fragments into apoptotic corpses, which are expulsed laterally into the follicular epithelium (taking <1 h). Engulfment by neighboring FCs only takes about one hour but full elimination of supernumerary PC material within the FCs takes several hours

This study has identified a highly-stereotyped process of developmentally-programmed epithelial cell extrusion with striking features (Figure 8). First, a long phase of cell remodeling (>7 h) with no signs of apoptosis or cell shrinkage. This phase involves the full envelopment of the PC to be eliminated by its PC neighbors, which then becomes isolated from follicle cells, germline cells and the basement membrane. We have thus termed this process cell ‘monosis’, for ‘isolation’ in Greek. Second, a quick apoptotic phase (<1 h) occurs. Strikingly, PC apoptotic corpses are expulsed laterally within the follicular epithelium only a few minutes after apical domain collapse of the PC to be eliminated. Finally, a third long (>7 h) clearance phase follows, during which apoptotic corpses are engulfed by neighboring FCs and fully eliminated over several hours.

Our results suggest a functional link between PC monosis and the activation of the apoptosis cascade. At least two mechanisms can be proposed to explain how the final steps of PC monosis, full envelopment by surrounding PCs, could trigger apoptosis. PCs to be eliminated transit from an initial state, characterized by equivalence with the other PCs and contacts with FCs, germline cysts and the basement membrane to a spherical shape upon becoming fully embedded between other PCs. This state may be sensed by the cell as a higher internal pressure, which could be translated into a signal for *hid* transcriptional activation. Similarly, mechanical pressure has been reported to induce specific gene transcription in the *Drosophila* embryo,^26^ and cell crowding can induce live cell extrusion in vertebrates via a stretch receptor signal transduction.^2, 3^ Alternatively, PCs, which become fully isolated upon monosis might not receive trophic/survival signals, thereby leading to apoptosis activation, in accordance with the trophic model for cell survival.^34^

The reinforcement of AJs between PCs during monosis may be important before extrusion. Similarly, active roles for AJs in tissue remodeling, including cell extrusion, have been reported.^28, 29^ During the later apoptotic shrinking phase, AJ components are lost in a caspase-dependent manner, which is in agreement with studies, indicating that AJ components are bona fide caspase substrates.^25, 30, 31, 32^

A parallel can be drawn between the cell monosis process and cases of cells wrapping around other cells in various biological processes. For instance, glial cells insulate axons to ensure fast and reliable transmission of action potentials.^33^ In the *Drosophila* pupal ommatidium, the four central cone cells become progressively surrounded laterally by two enwrapping primary pigment cells, which isolate them from secondary and tertiary pigment cells.^34^ In *Drosophila* late stage follicles, the surviving PC pair will be surrounded by a group of 6–8 FCs (border cells), and then be transported passively at the center of the group by the border cells that adopt invasive migratory properties.^22, 35^ Cell monosis as a preparation for epithelial cell extrusion has not been described elsewhere. It is possible that this is because other extrusion events within complex tissues have not been characterized with sufficiently high-resolution 3D reconstruction and 4D imaging.

Cases of both apical and basal epithelial cell extrusion have been reported,^5, 6, 7^ but PC extrusion is lateral within the epithelium, which is to our knowledge the first example of such a process. However, we expect this mode of extrusion not to be unique to PCs, but to depend on specific tissue constraints. In the case of PCs, for example, pressure may be exerted apically from the growing germline cyst and basally from the presence of the basement membrane, leading to lateral extrusion. Further study of cell extrusion and tissue remodeling events occurring in human gastrointestinal or ovarian epithelial tissues, where cells are intensely renewed, would be of great interest. This would allow testing the universality of cell monosis and lateral cell extrusion and could provide important insights for future cancer drug development.

The live imaging system we developed also clearly revealed the fate of the dying supernumerary PCs. Some apoptotic corpses were engulfed and completely digested by immediately adjacent FCs, while other corpses travelled further away within the epithelium to be engulfed by more distant FCs. This confirms that FCs have proficient clearance properties since they are also responsible for the uptake of nurse cell and oocyte remains upon starvation-induced egg chamber degeneration.^36^

Finally, our results indicate that JAK/STAT signaling is required for the stereotyped cell remodeling events (cell monosis) that always occur prior to apoptosis. We consider that cell monosis is thus a prerequisite to activate the apoptotic cascade in the supernumerary PCs, leading to lateral extrusion of apoptotic corpses (Figure 8). Indeed, there is no expression of apoptotic markers during the long cell monosis phase and blocking the apoptotic cascade did not block the early cell monosis events but rather the later formation of apoptotic corpses for expulsion. In contrast, PC monosis was inhibited upon reduction of JAK/STAT signaling such that largely unremodeled PC groups with supernumerary PCs showing no signs of apoptosis were maintained until at least stage 10 of oogenesis (40 h longer than in controls). These results therefore support a model in which the JAK/STAT pathway is active, whereas the apoptotic cascade is inactive, during primary PC remodeling events. Later, caspase-dependent downregulation of AJ components possibly facilitates the extrusion of PC apoptotic corpses.

In recent years, the highly conserved tumorigenic JAK/STAT pathway has been implicated in epithelial morphogenesis both in *Drosophila* and in mammals for the development of many different organs.^13, 14, 35, 37, 38, 39^ However, the cellular and molecular mechanisms underlying these processes and the specific target genes involved remain largely unknown. Future work on JAK/STAT signaling in PCs and surrounding FCs will address these questions.

## Materials and methods

### *Drosophila* stocks and genetics

Strains carrying the following transgenes were used in this study: *UAS-mCD8:GFP* (chr. II), *UAS-p35* (chr. II), *UAS-Par3:GFP* (kindly provided by Antoine Guichet), P (PZ) *hid*^*0514*^ (Hid-lacZ enhancer trap), *ECad:GFP*^*KI*^ (knockin^40^), *ubi-PH:mChFP* (chr. II) (kindly provided by Y Bellaiche). Strains carrying RNAi constructs from VDRC (Vienna, Austria) collections were *UAS-upd-RNAi* (3282) and *UAS-stat92E-RNAi* (43866). The *upd-Gal4*^19^ and *fruitless-Gal4*^20^ enhancer traps were used to specifically target PCs and FCs, respectively. TubP-Gal80^ts^ (chr. II) (kindly provided by Jacques Montagne) was used to avoid embryonic lethality associated with expression of *UAS-Stat92E-RNAi* in FCs. All crosses targeting PCs were conducted at 25 °C until mid-pupariation. Then, they were placed at 29 °C and dissected 4 or 5 days after eclosion. For crosses involving Gal80^ts^, they were conducted at 18 °C until eclosion and then, shifted to 29 °C until dissection four or five days later.

### Immunohistochemistry

Ovary dissections were performed according to^20^ except that Triton 0.3X was used instead of Tween-20 and nuclei were stained with DAPI (1 ng/ml final concentration). The following primary antibodies were used: mouse monoclonal anti-Fas3 (1:20, DSHB, 7G10), rat anti-ECad (1:50, DSHB, DCAD2), rabbit anti-Par3 (1:500, Andreas Wodarz), rabbit anti-GFP (1:500, Interchim), rabbit anti-human cleaved caspase 3 (1:20, Ozyme), guinea pig polyclonal anti-Hid (1:100, a gift from D Ryoo, New York University, USA), mouse monoclonal anti-Diap1 (1:200, B Hay laboratory, California Institute of Technology, Pasadena, CA, USA), rabbit polyclonal anti-*β*-galactosidase (1:200, MP Biomedicals, Santa Ana, CA, USA). The Alexa Fluor anti-rabbit (488) and anti-rat (647) secondary antibodies (Molecular Probes, Eugene, OR, USA) were used at 1:200 and anti-mouse-Cy3 (Jackson Immunoresearch, West Grove, PA, USA) was used at 1:100.

### Microscopy and figure montages

Confocal images in Figures 2,3,4,5 were captured with a Leica TSC SP8 microscope using LAS X software (Leica, Wetzlar, Germany). Confocal images in Figures 1,6 and 7 and Supplementary Figures 1 and 2 were taken with a Nikon eclipse TE 2000-U microscope using the EZ-C1 3.30 software (Nikon, Tokyo, Japan). Confocal images stacks were exported in original format and processed using Fiji, an image processing package - a "batteries-included" distribution of ImageJ, bundling a lot of plugins which facilitate scientific image analysis. All images were then processed further using Adobe Photoshop CS5 software (Adobe Systems Canada, Ottawa, ON, Canada).

### Egg chamber culturing and live imaging

In order to address the dynamics of the PC remodeling events in real time, we adapted a system for live imaging that was initially designed for late ovarian follicles.^41^ We established culture conditions allowing imaging of young follicles over long periods of time (most often about 6 h, but in rare cases up to 10 h), which was essential since supernumerary PC elimination turned out to be a lengthy process. The number of movies obtained was limited by the fact that young follicles in ovariole cultures were infrequently positioned appropriately for real-time imaging (see Figure 1a). Also due to the length of time needed for PC elimination and the fact that small young follicles (<50 *μ*m diameter) were not always stationary, the entire sequence of events was captured on separate films, each covering overlapping parts of the full sequence. In order to preserve stages 3–4 egg chambers, extraction was performed by anteriorly pulling stage 9–10 egg chambers. Removal of the muscular sheath enveloping the ovariole was a critical step. Its gentle disruption was performed using a tungsten needle. Dissected ovarioles were transferred onto a micro-glass slide and mounted in 50 *μ*l of medium containing insulin (40 *μ*g/*μ*l) on a Lumox dish (Sarstedt, 946077410) sealed with carbon oil, to allow gas exchanges. Confocal image stacks of stage 3–4 egg chambers with more than two PCs were acquired for several hours. Due to the location of PCs at egg chamber poles, and the fact that successive egg chambers are connected to each other at the poles, mounted ovarioles most often presented PCs with the apical-basal axis parallel to the XY plane of view from the objective (objective 1, Figure 1a). Therefore imaging of the apical domain of the PCs over time necessitated 3D renditions of deep stack acquisitions of the follicles with 0.3<z step <0.5 *μ*m. Since imaging over long periods greatly affects fluorescent signals, this type of acquisition did not allow filming of PCs over sufficient durations. However, some follicles were oriented such that the apical-basal axis of the PCs was along the XZ plane of view from the objective (objective 2, Figure 1a), greatly facilitating stack acquisitions. For Figure 3, eight films of different durations each separated by 5 min were concatenated. Image stacks of 20 slices (0.5 *μ*m z steps) were acquired at 5 min time intervals. For Figures 4 and 5, image stacks of 10 slices (1 *μ*m z steps) were taken every 1.5 min.

### Image treatment using FIJI software

Confocal image stacks were processed with FIJI software (All platforms, no JREs). Reslices and 3D renditions, necessary for certain orientations of the PC group (objective 1, Figure 1a), were made in order to determine area and shape of PCs except when PC orientation did not require them (objective 2, Figure 1a). Z slice projections were of 1 slice (Figures 4 and 5, z step: 1 *μ*m), 2–3 consecutive slices (Figures 1 and 2, Figures 6 and 7, z step: 0.2–0.5 *μ*m) and 5 consecutive slices (Figure 3 z step: 0.5 *μ*m). 3D projections were of 30–40 consecutive slices (Figures 1 and 7, z steps: 0.2 *μ*m). For Figure 3, the focal plane with the highest intensity of ECad:GFP signal (at the subapical adherens junction domain) was selected for each image stack at all time points and a (+2/−2) sub-stack was extracted using a macro written and kindly provided by Y. Bellaïche. Resulting five image stacks were projected using SUM slices. To compensate for global tissue movements and make PC tracking simpler, we used the Fiji plug-in ‘StackReg’.

### PC shape quantifications

Image stacks of supernumerary PC groups were cropped and treated if necessary either for reslicing or 3D rendering in order to determine their respective shapes in the apical domain using E-Cadherin:GFP accumulation. 4SP-2SP nomenclatures were used for supernumerary PCs with ≥4 to two contacts with other cells (including the contacts with two PCs contacts). Supernumerary PCs were considered detached when the E-Cadherin:GFP-positive lens-shaped 2SP structure was found beneath an apical contact between enveloping PCs. Apical domain area measurements were made from image stack reslices or 3D renditions using the freehand selection in Fiji. In Figures 2e, 2f and 7c, surface areas were calculated for 3PC groups, as the mean of three different measures on the supernumerary PCs and the surrounding 2PCs.

### Statistical analysis

For Figures 1i–i’,Figures 2e and 7c normal distribution was tested with Lilliefors (Kolmogorov-Smirnov) normality test. One-way ANOVA post-hoc Tukey contrasts were performed to compare sample means. For Figure 6k, E-Cadherin:GFP sample mean intensities were compared using Welch’s *t*-test. All statistical analysis was performed with R version 3.2.1. Significance for the statistical test was coded in the following way based on the *P*-values: *0.01<*P*<0.05; **0.001<*P*<0.01; ***0<*P*<0.001.
