# Supplementary material for: JAK/STAT signaling is necessary for cell monosis prior to epithelial cell apoptotic extrusion
Source: Cell Death Dis. 2017 May 25;8(5):e2814–. doi: 10.1038/cddis.2017.166 (PMC5520696; doi:10.1038/cddis.2017.166)
Supplement: Supplementary Legends [file cddis2017166x9.pdf]

Supplementary Movie legends

Supplementary Movie 1. 3D reconstruction animations of PC groups during the remodeling phase. Entire confocal stacks of stage 3-4 wild-type follicles immunostained for Fasciclin 3, accumulating at high levels at PC-PC interfaces, were used for 3D reconstruction of PC groups at the poles of these follicles viewed from the top within the plane of the follicular epithelium (see objective 2 in Figure 1a). A royal color code was used to highlight Fas3 protein concentrations on a scale from lowest (blue) to highest (white). Four configurations suggest a stereotyped sequence of remodeling events beginning with the initial state, followed by partial envelopment of the supernumerary PC by the two other PCs, then full lateral envelopment and finally apical detachment. Tilted animations allow highlighting of the envelopment of the supernumerary PCs by the two neighboring PCs.

Supplementary Movie 2. Animations through the entire volume of two groups of 3 PCs at the initial and full lateral envelopment states during the remodeling phase. Animations of confocal image stacks through the apical-basal axis of groups of 3 PCs from wild-type stage 3-4 follicles immunostained for E-Cad:GFP and Fas3. The group of 3 PCs shown in the left column is at the initial state and that in the right column at full lateral envelopment. Top: merge of ECadh:GFP (green) and Fas3 (red) immunostainings. Center: ECadh:GFP immunostaining. Bottom: Fas3 immunostaining. Frames 1, 59 and 112 are at the basal-most level, frames 29 and 85 at the apical-most level. Scale bar: 5 µm.

Supplementary Movie 3. Supernumerary PC remodeling at the subapical E-Cad domain. The frames in Figure 3a-c are taken from this time-lapse acquisition. Each frame corresponds to a projection of three consecutive 0.3µm confocal slices. Full length of acquisitions: 310 min. Interval between acquisitions: 5 min. Frames per second: 7.

Supplementary Movie 4. Cell remodeling of supernumerary PCs followed by lateral extrusion of apoptotic corpses. The frames in Figure 4a-h are taken from this time-lapse acquisition. Single confocal slices (1 μm) at the subapical level for ECadh:GFP and at the lateral level for PH:chFP. Full length of acquisitions: 190 min 30 sec. Interval between acquisitions: 1 min 30 sec. Frames per second: 7.

Supplementary Movie 5. Engulfment and digestion of PC apoptotic corpses by FCs. The frames in Figure 5a-f are taken from this time-lapse acquisition. Single confocal slices (1 μm) at the lateral level through PCs and FCs visualized by the fusion protein PH:chFP.

Supplementary Movie 6. Hid protein is only detected in fully enveloped, apically detached, spherical PCs. Animation along the apical-basal axis (left) and pivoting of a 3D reconstruction (right) of the PC group in Figure 6b' immunostained for Fas3 (red) and Hid (green).

Supplementary Movie 7. Animation along the apical-basal axis of a group of 3 PCs from a late stage follicle with reduced JAK/STAT signaling. An apical-basal animation through the group of 3 PCs of the stage 7 follicle in Figure 7b,b'. At this late stage remodeling of the supernumerary PC is blocked at the initial state. Left: merge of ECadh:GFP and Fas3 immunostainings. Center: ECadh:GFP immunostaining. Right: Fas3 immunostaining. Apical-most frames are 1, 51, and 100, basal-most frames are 27 and 76. Scale bar: 5 μm.

Supplementary Movie 8. 3D reconstruction animations of 3 PC groups of a late stage JAK/STAT-depleted follicles blocked at early remodeling states. Pivoting animations of two groups of 3 PCs of the same genotype as in Figure 7f to emphasize the block in apical detachment upon inhibition of JAK/STAT signaling.
