# Supplementary figures and images for: JAK/STAT signaling is necessary for cell monosis prior to epithelial cell apoptotic extrusion
Source: Cell Death Dis. 2017 May 25;8(5):e2814–. doi: 10.1038/cddis.2017.166 (PMC5520696; doi:10.1038/cddis.2017.166)

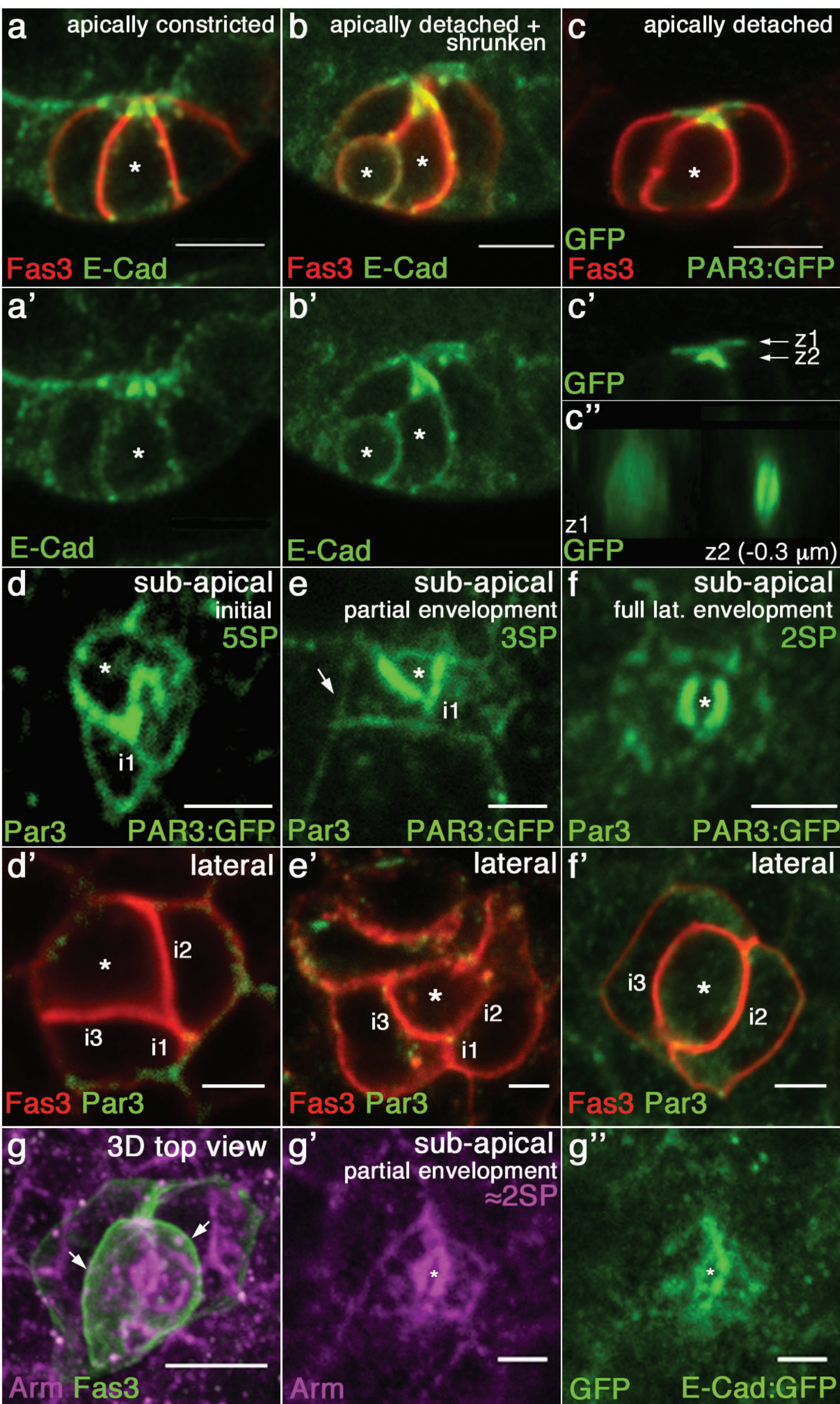

Supplement: Supplementary Figure 1 [file cddis2017166x10.pdf]

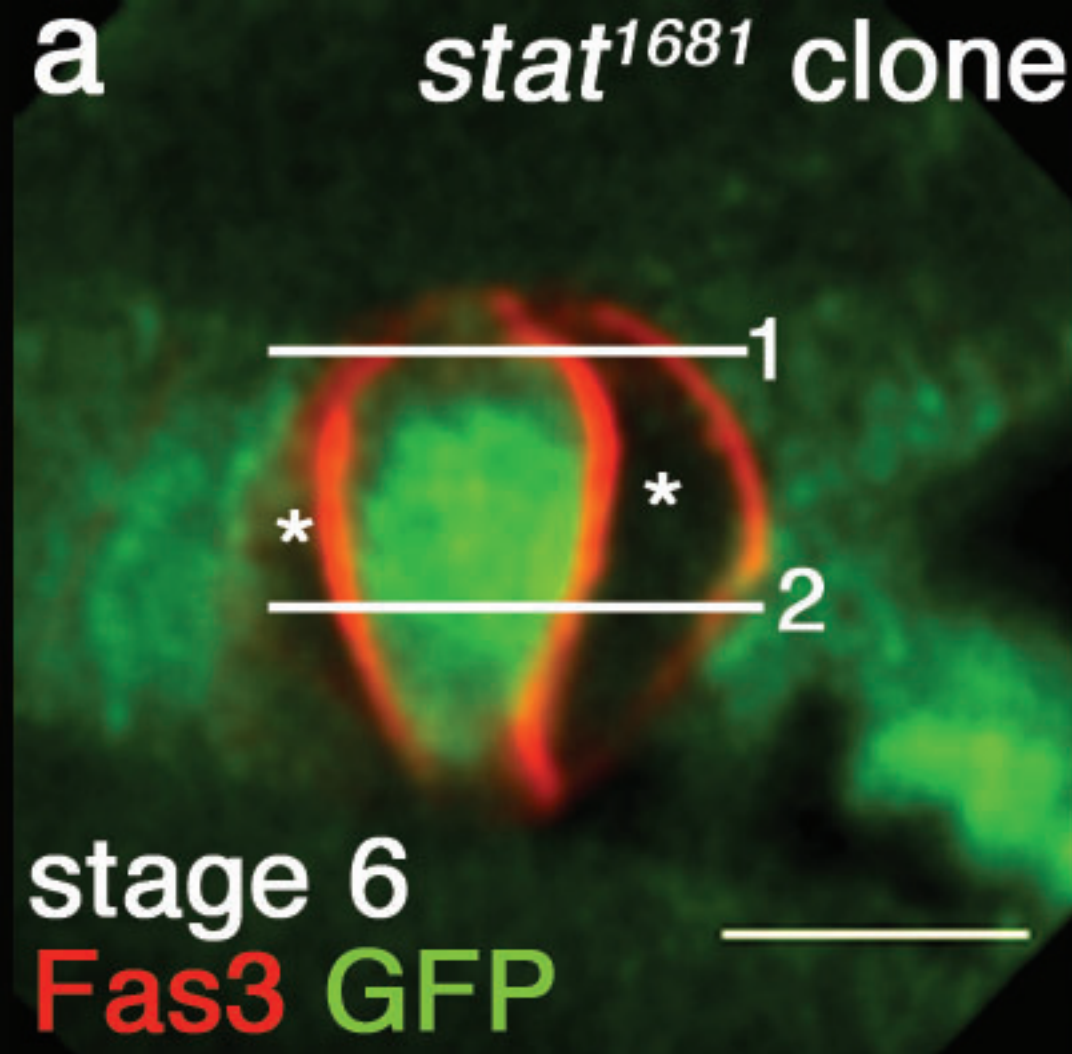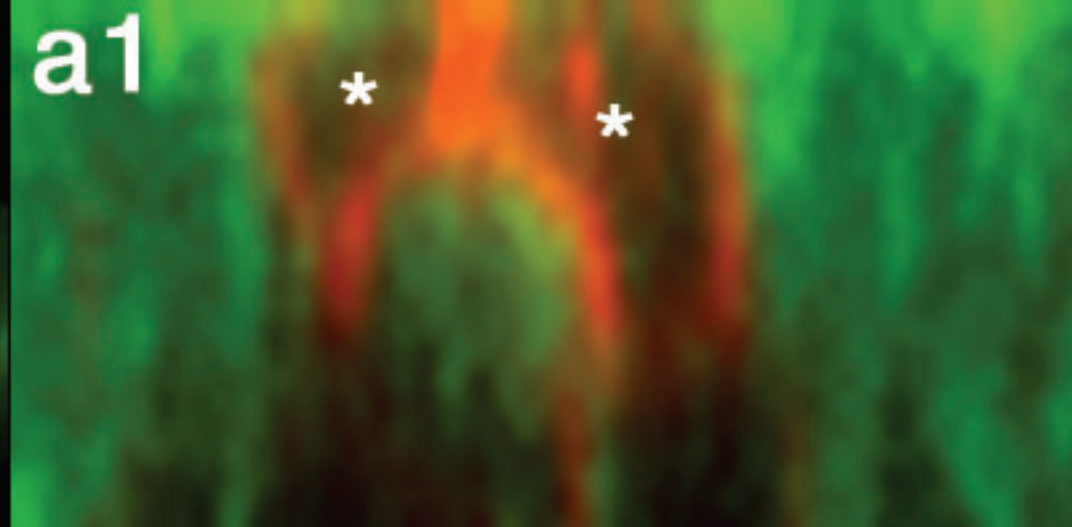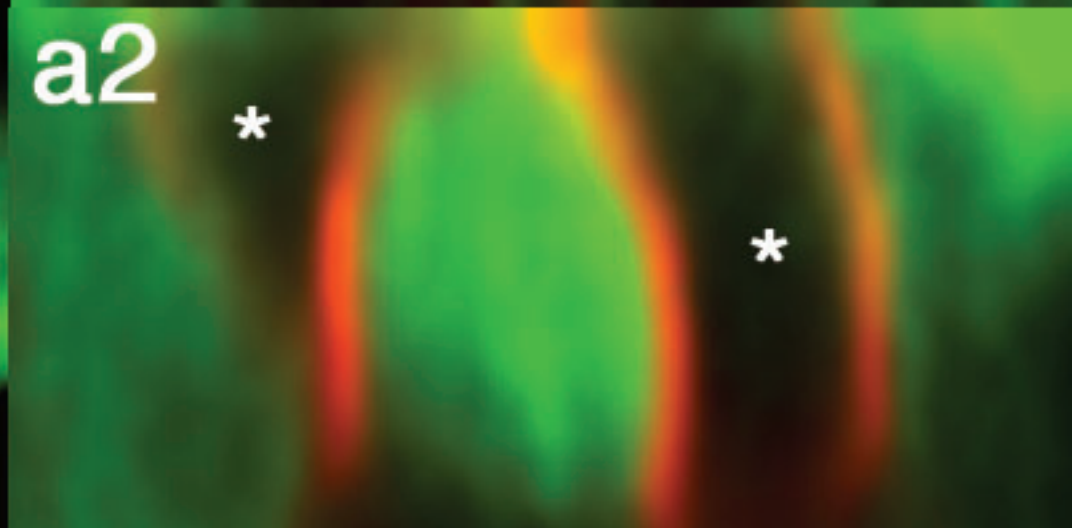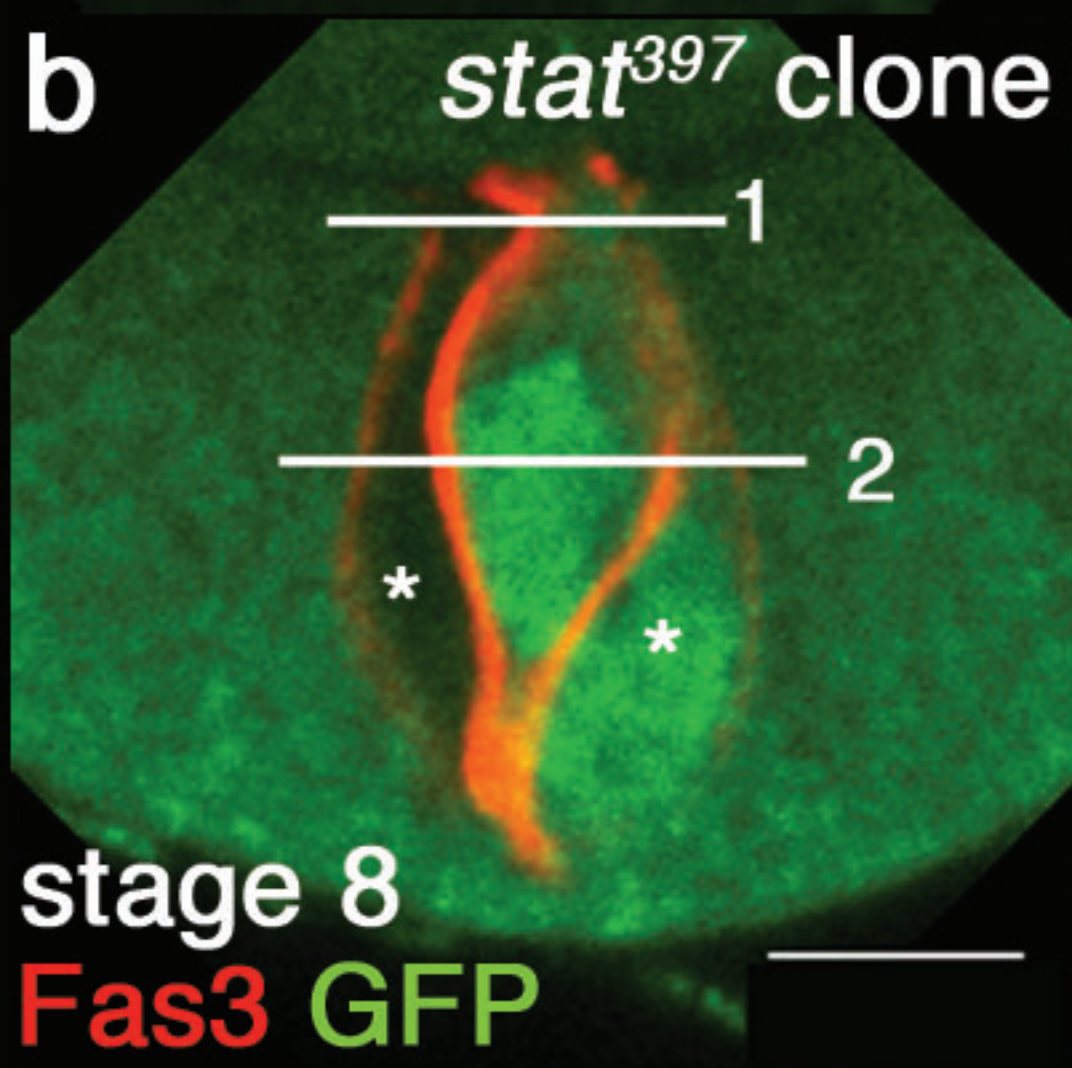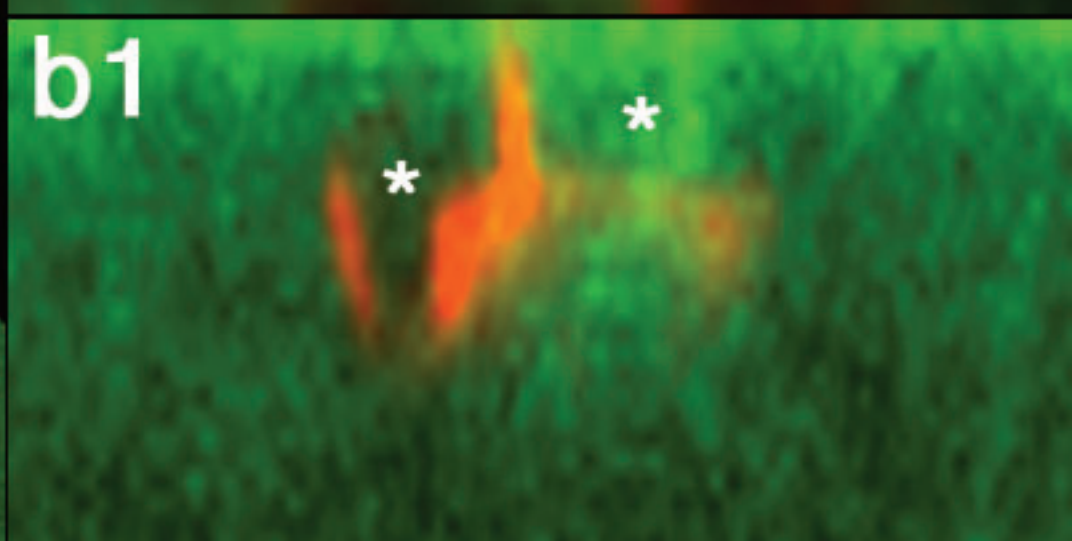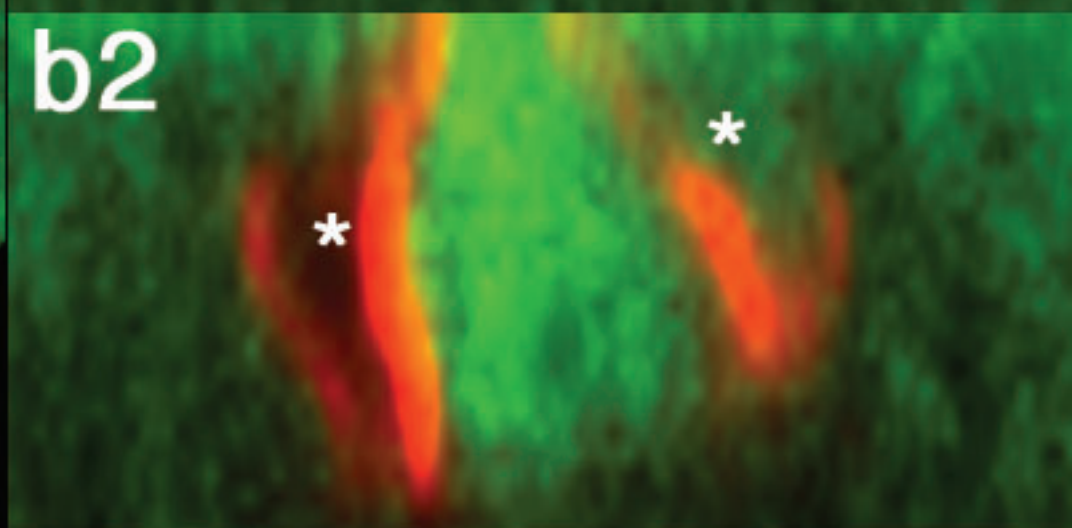

Supplement: Supplementary Figure 2 [file cddis2017166x11.pdf]

a

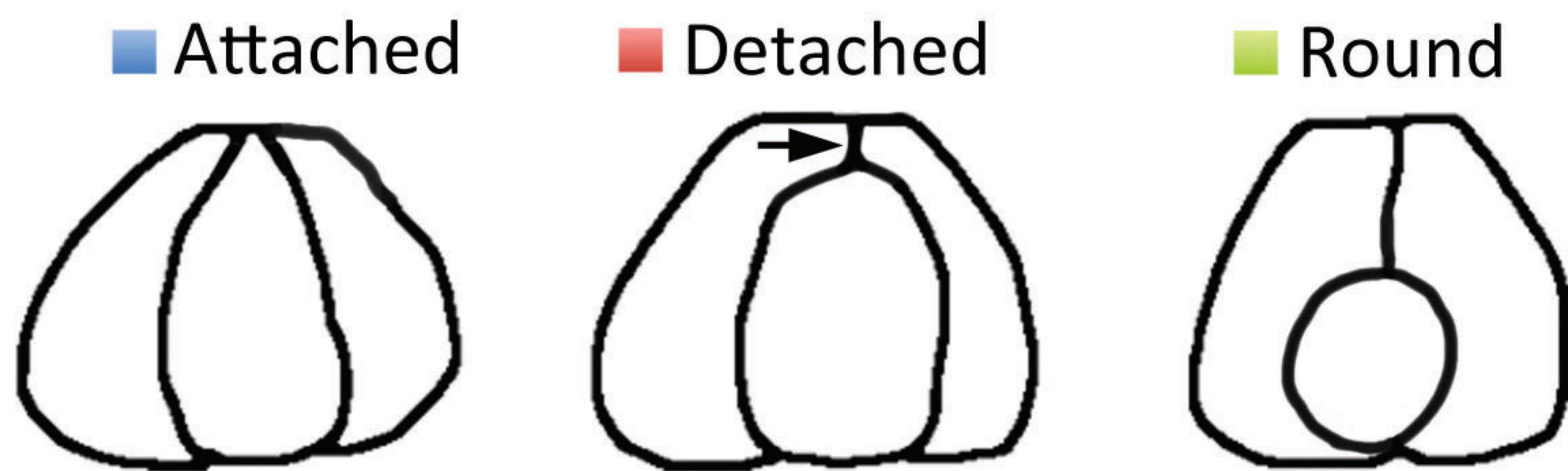

b

### Distribution of cell shapes in 3 PC groups

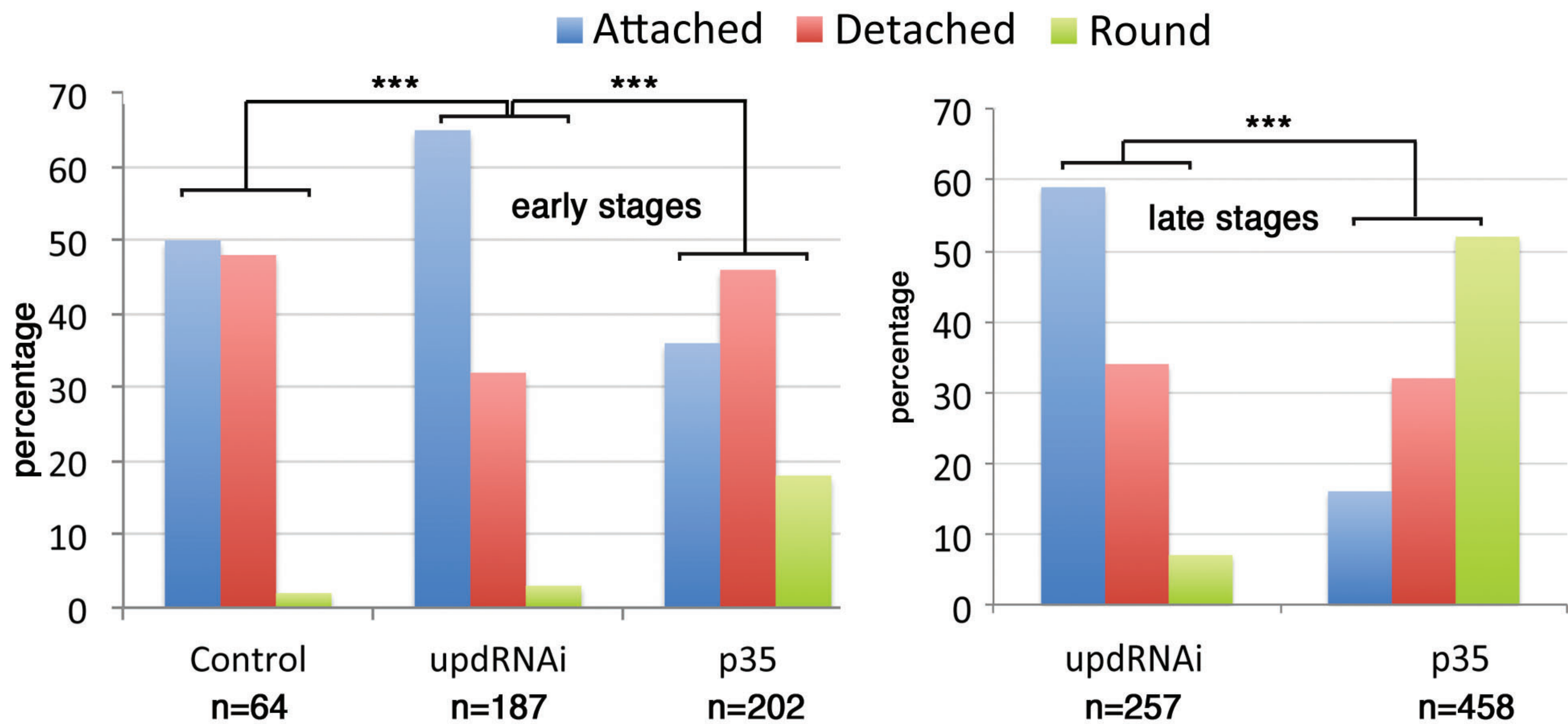

c

### Distribution of cell shapes in 4 PC groups

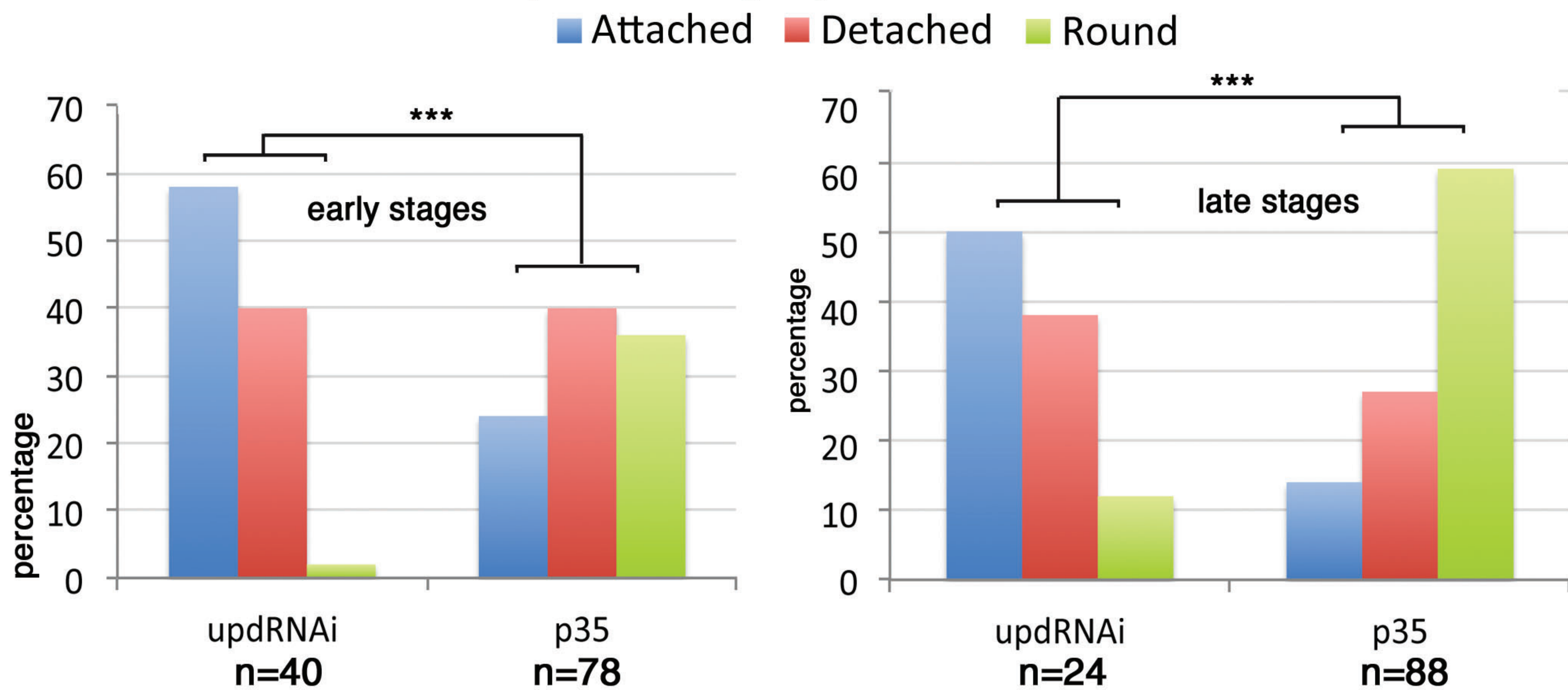

Supplement: Supplementary Figure 3 [file cddis2017166x12.pdf]
