# Supplementary material for: JAK/STAT signaling is necessary for cell monosis prior to epithelial cell apoptotic extrusion
Source: Cell Death Dis. 2017 May 25;8(5):e2814–. doi: 10.1038/cddis.2017.166 (PMC5520696; doi:10.1038/cddis.2017.166)
Supplement: Supplementary Figure 4 [file cddis2017166x13.pdf]

Supplementary figure legends

Supplementary Figure 1. Accumulation of adherens junction components at PC-PC interfaces. All follicles presented are at stages 3-4. (**a,b,c**) 3PC (**a,c**) and 4 PC (**b**) groups from confocal slice projections taken from side views (see objective 1 in Figure 1a) immunostained for Fas3 (red) to mark PC membranes and for E-Cad (green) and GFP (green) to detect Par3:GFP targeted in PCs (with *the upd-Gal4* driver). Remodeling states of the supernumerary PCs (asterisks) are indicated. (**a'-c'**) Indicates strong accumulation of these two adherens junction components sub-apically. (**c''**) Orthogonal reslices of (**c'**). z1 presents the apical surface of the two enveloping PCs, while z2 is a slice that is 0.3µm lower than z1 where the presence of a lens-shaped Par3:GFP domain of the supernumerary PC indicates its apical detachment. (**d-f**) Projections of sub-apical and (**d'-f'**) lateral confocal slices taken from a top view (see objective 2 in Figure 1a) of 3PC-containing groups at the indicated steps of the remodeling phase immunostained for Fas3 and Par3:GFP (as in **a-c**). Par3:GFP accumulates at high levels at the PC-PC interfaces of the supernumerary PC (asterisks) and delimits the same subapical domain polygonal shapes (**d-f** - 5SP, 3SP and 2SP) as ECad (Figure 2). The i1,i2,i3 interfaces are the same as those defined in Figure 1. (**g-g"**) E-Cad:GFP knock-in expressing 3PC-containing group immunostained for Armadillo/β-Catenin (Arm-magenta), GFP and Fas3 (green). (**g**) 3D reconstruction of confocal image stack taken from a top view (see objective 2 in Figure 1a). Arrows point to the interfaces between the enveloped supernumerary PC and the two enveloping PCs. (**g'**) Sub-apical confocal slice from (**g**) with an asterisk marking the sub-apical domain accumulating Arm in a 2SP or lens shape as E-Cad in Figure 2). Scale bars: 5 µm, except e,e',g',g": 2.5 µm. Genotypes (a,b): *upd-gal4/+*. (c-f): *upd-gal4/+; UAS-Par3:GFP*; (g-g''): *upd-gal4/+; E-Cad:GFP/+*.

Supplementary Figure 2. JAK/STAT activity is required in surrounding PCs for envelopment of the supernumerary PCs. (**a,b**) Confocal slice projections taken from a side view (see objective 1 in Figure 1a) of 3PC-containing groups at late stages of oogenesis (stages 6 and 8) identified by Fas3 membrane immunostaining and containing $stat92E^{1681}$ *and* $stat92E^{397}$ null mutant clones marked by absence of GFP immunostaining. Since none of the PCs in each group show apical detachment at these late stages, then the presence of *stat92E* mutant PCs leads to a block in this remodeling event. (**a1-a2**, **b1-b2**) Orthogonal sections corresponding to the levels indicated by horizontal lines 1 and 2 in (**a,b**) indicate that in each case the supernumerary PC is partially enveloped by the other two PCs (asterisks). Therefore, the presence of *stat92E* mutant PCs has blocked the remodeling process of these groups of 3 PCs early, during the enveloping process.

Supplementary Figure 3. Quantification of the effects of JAK/STAT and caspase inhibition on supernumerary PC remodeling. (**a**) Schematic drawings of 2D side views (see objective 1 in Figure 1a) of 3PC-containing groups with the supernumerary PCs in attached, apically detached or round/shrunk configurations defined by a color code to be used in graphs. Arrow points to the newly-formed contact between the two surviving PCs upon apical detachment of the supernumerary PC. (**b**,**c**) Percentage of supernumerary PCs shapes in early stages 3-5 and late stages 6-10 of oogenesis in 3PC- (**b**) and 4PC- (**c**) containing groups of the following genotypes: Control: *upd-gal4/+*. upd-RNAi: *upd-gal4/+; UAS-upd-RNAi/+*. p35: *upd-gal4/+; UAS-p35/+*. n represents the number of groups with supernumerary PCs analyzed. The graphs show that groups with supernumerary PCs are blocked in earlier remodeling phases when the JAK/STAT pathway is inhibited than when apoptosis is inhibited. *** indicates the χ^2 test p values for the compared distributions indicated with brackets (see Supplementary Materials and Methods).
