# Supplementary material for: JAK/STAT signaling is necessary for cell monosis prior to epithelial cell apoptotic extrusion
Source: Cell Death Dis. 2017 May 25;8(5):e2814–. doi: 10.1038/cddis.2017.166 (PMC5520696; doi:10.1038/cddis.2017.166)
Supplement: Supplementary Materials and Methods [file cddis2017166x14.pdf]

Supplementary materials and methods

Armadillo immunostaining

Ovary dissections were performed as described in Materials and Methods. Immunostaining was performed as described in Materials and Methods with the following modifications: Ovaries were incubated overnight at 4°C with agitation in primary antibodies, mouse anti-Fasciclin 3 (1:20-DSHB) and rabbit anti-GFP (1:500-DSHB) to detect E-Cadherin:GFP, followed by staining with secondary antibodies using Alexa Fluor anti-rabbit (488) at 1:200 and anti-mouse-Cy3 at 1:100 for two hours. Then another round of immunostaining including a new blocking step, primary antibody incubation with mouse anti-Armadillo (1:400, DSHB) overnight at 4°C with agitation and secondary antibody incubation with Alexa Fluor anti-mouse (647) at 1:200 for two hours was performed.

Mosaic analyses

Stat92E mutant clones were generated through mitotic recombination by the FLP/FRT technique (Xu and Rubin, 1993). Females of the following genotypes were analyzed: hs-*flp*/+; FRT82B-GFP/FRT82B-*Stat92E*^{397} and hs-*flp*/+; FRT82B-ubiGFP/FRT82B-*Stat92E*^{1681}. Mitotic clones, marked by the absence of GFP, were induced by two 1-hour heat shocks at 38°C at mid-pupae and 24 hours before dissection. Adult females were dissected 4-5 days after eclosion.

Statistical analysis

Statistical significance of cell shape distributions was assessed by X^2 test between control and *upd*-RNAi, control and *p35*, and *upd*-RNAi and *p35* conditions. *** correspond to $p<0.001$.
